# Structural Effects in Lithiocuprate Chemistry: The Elucidation of Reactive Pentametallic Complexes

**DOI:** 10.1002/chem.201304824

**Published:** 2014-02-19

**Authors:** Philip J Harford, Andrew J Peel, Joseph P Taylor, Shinsuke Komagawa, Paul R Raithby, Thomas P Robinson, Masanobu Uchiyama, Andrew E H Wheatley

**Affiliations:** [a] Department of Chemistry, University of CambridgeLensfield Road, Cambridge, CB2 1EW (UK); [b] Advanced Elements Chemistry Research Team, RIKEN Center for Sustainable Resource Science (CSRS) and Elements Chemistry LaboratoryRIKEN, 2-1 Hirosawa, Wako-shi, Saitama 351-0198 (Japan), Graduate School of Pharmaceutical Sciences, The University of Tokyo, 7-3-1 Hongo, Bunkyo-ku, Tokyo 113-0033 (Japan); [c] Department of Chemistry, University of Bath, Claverton Down BathBA2 7AY (UK)

**Keywords:** copper, density functional calculations, directed metalation, lithium, solid-state structures

## Abstract

TMPLi (TMP=2,2,6,6-tetramethylpiperidide) reacts with Cu^I^ salts in the presence of Et_2_O to give the dimers [{(TMP)_2_Cu(X)Li_2_(OEt_2_)}_2_] (X=CN, halide). In contrast, the use of DMPLi (DMP=*cis*-2,6-dimethylpiperidide) gives an unprecedented structural motif; [{(DMP)_2_CuLi(OEt_2_)}_2_LiX] (X=halide). This formulation suggests a hitherto unexplored route to the in situ formation of Gilman-type bases that are of proven reactivity in directed *ortho* cupration.

Organocuprate(I) complexes in general,[1] and amidocuprates in particular, have proved to be tremendously important in effecting stereoselective organic transformations,[2] with recent work yielding lithiocuprates of the type RR′CuXLi_2_ (R, R′=organyl, TMP (TMP=2,2,6,6-tetramethylpiperidide); X=CN, halide) and the new field of directed *ortho* cupration (DoC). It has been noted that the TMP group could react to achieve chemoselective DoC transformations and the subsequent trapping of electrophiles or oxidative ligand coupling showed the significant potential of these reagents in C=C and C=O bond formation.[3], [4]

Structural organocuprate(I) chemistry was recently the subject of review.[5] Recent advances have revealed so-called Gilman-type monomers and dimers[6] and (in line with theory)[7] heteroleptic monomers[8], [9] and dimers (Figure 1 a, b, d).[10] Conversely, cyanide-containing Lipshutz cuprates[11] with heteroaggregate structures have now been elucidated (Figure 1 e),[3] with very recent work proving that the replacement of X=CN by X=halide affords structurally analogous complexes,[4c,d, 6] and suggesting use of the term Lipshutz-type to describe this wider group of comparable cuprates. Interestingly, although the reactivity of Lipshutz-type cuprates has been considered to often exceed that of their Gilman-type counterparts,[4b, 8, 13] it was recently suggested that a Lipshutz-type reagent could be used to generate a *more reactive* Gilman-type intermediate in situ.[6]

**Figure 1:**
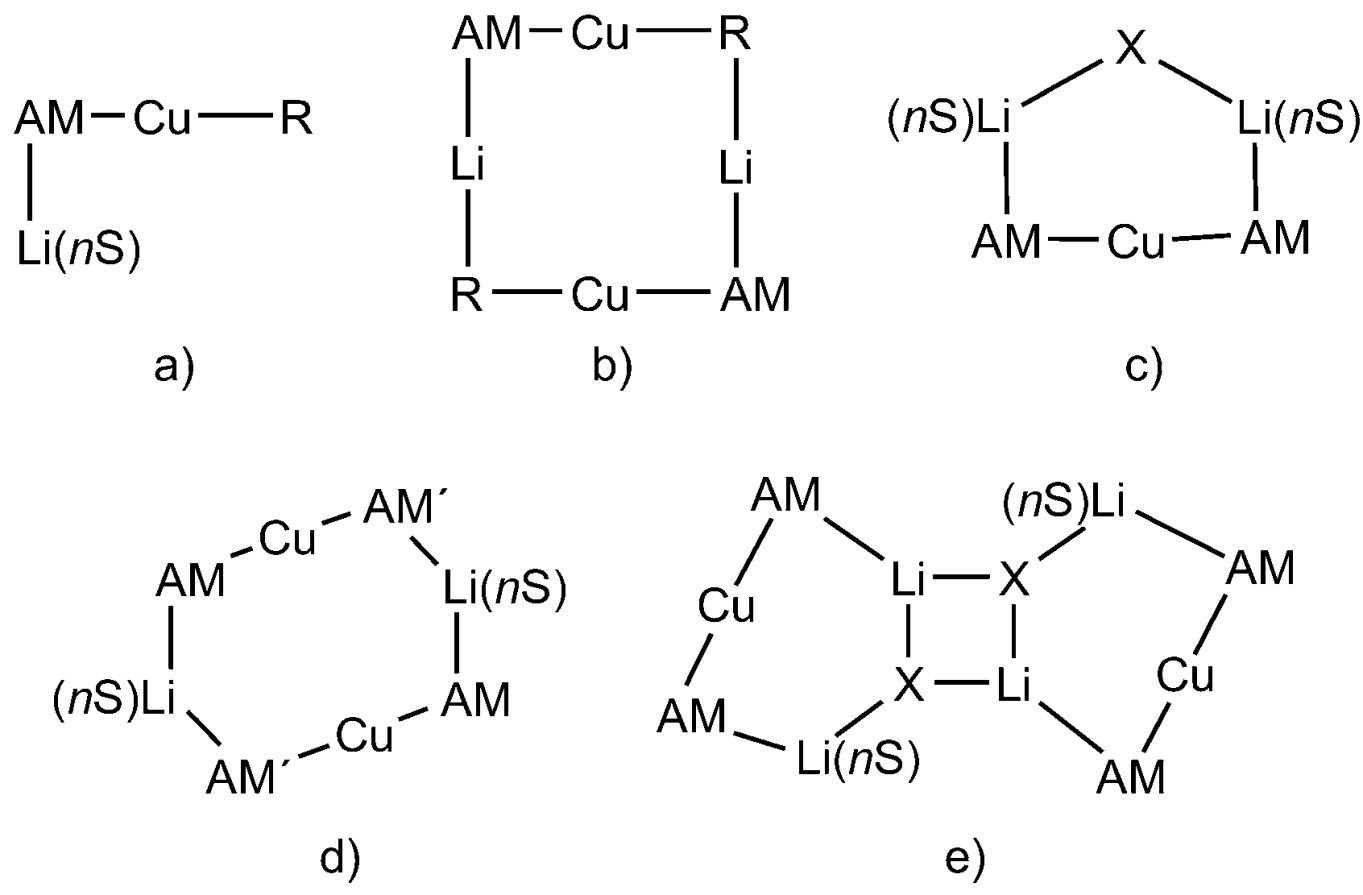
Established lithium amidocuprate structure types; a) AM=N(CH_2_Ph)Et, R=Mes, *n*=3, S=THF;[9] AM=TMP, R=Ph, *n*=3, S=THF;[8] AM=TMP, R=Me, *n*=1, S=TMEDA;[8] b) AM=N(CH_2_Ph)_2_, R=Mes;[10] c) AM=X=NPh_2_, *n*=1, S=OEt_2_;[12] d) AM=NHMes, AM′=NHPh, *n*=1, S=DME;[12] e) AM=TMP, X=CN, Cl, Br, I, *n*=1, S=THF.[3, 4b–d, 6]

Presently we report the investigation of ligand effects through varying the amido component of new lithiocuprate bases. Data reveal a hitherto unrecognised class of cuprate heteroaggregate.

We have recently sought to develop our understanding of ligand and solvent influences on lithiocuprate structures by modifying our previous syntheses of [{(TMP)_2_Cu(X)Li_2_(thf)}_2_] (X=CN,[3] halide[6]). To this end, TMPLi was added to CuCN before introducing Et_2_O. Storage of the resulting solution afforded crystalline **1**, the X-ray diffraction of which showed Lipshutz cuprate [{(TMP)_2_Cu(CN)Li_2_(OEt_2_)}_2_]. Though the quality of the data were poor (*R*_int_>10 %), the connectivity was unambiguous and the dimer was plainly analogous to that seen with THF.[3] Comparable reactions using CuHal gave [{(TMP)_2_Cu(Hal)Li_2_(OEt_2_)}_2_] (Hal=Cl **2**, Br **3**, I **4**), establishing the general isolation of the structure-type seen for **1** (Scheme 1). In each case superior crystal data were obtained, with structural parameters found to be closely related to those seen in [{(TMP)_2_Cu(Hal)Li_2_(thf)}_2_] (Figure 2).[4c,d, 6] The monomeric Lipshutz-type building blocks in **2**–**4** revealed symmetrical 6-membered rings with each amide acting as an intermetal linker through the construction of uniform Cu-N-Li bridges (Cu-N-Li 90.4(2)-92.26(12)°, Cu-N-Li(OEt_2_) 89.1(3)-91.8(2)°).

**Scheme 1:**
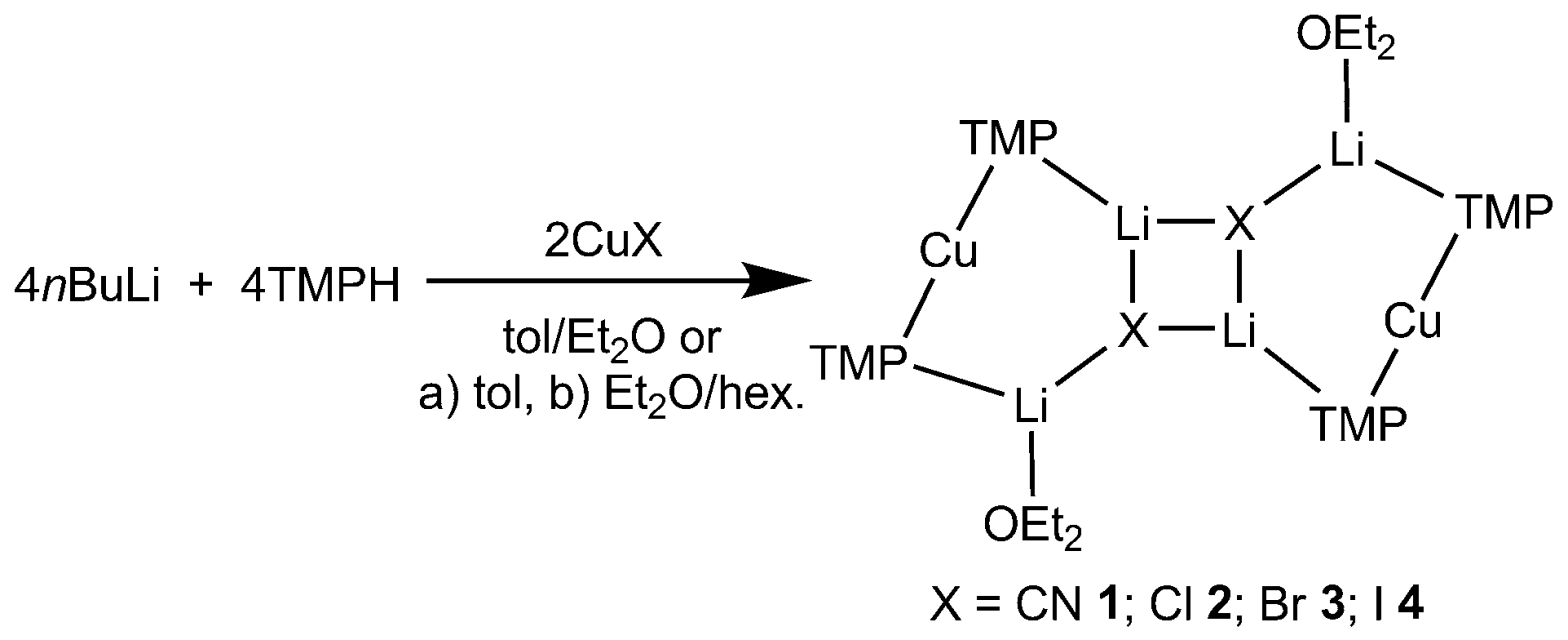
Synthesis of 1–4.

**Figure 2:**
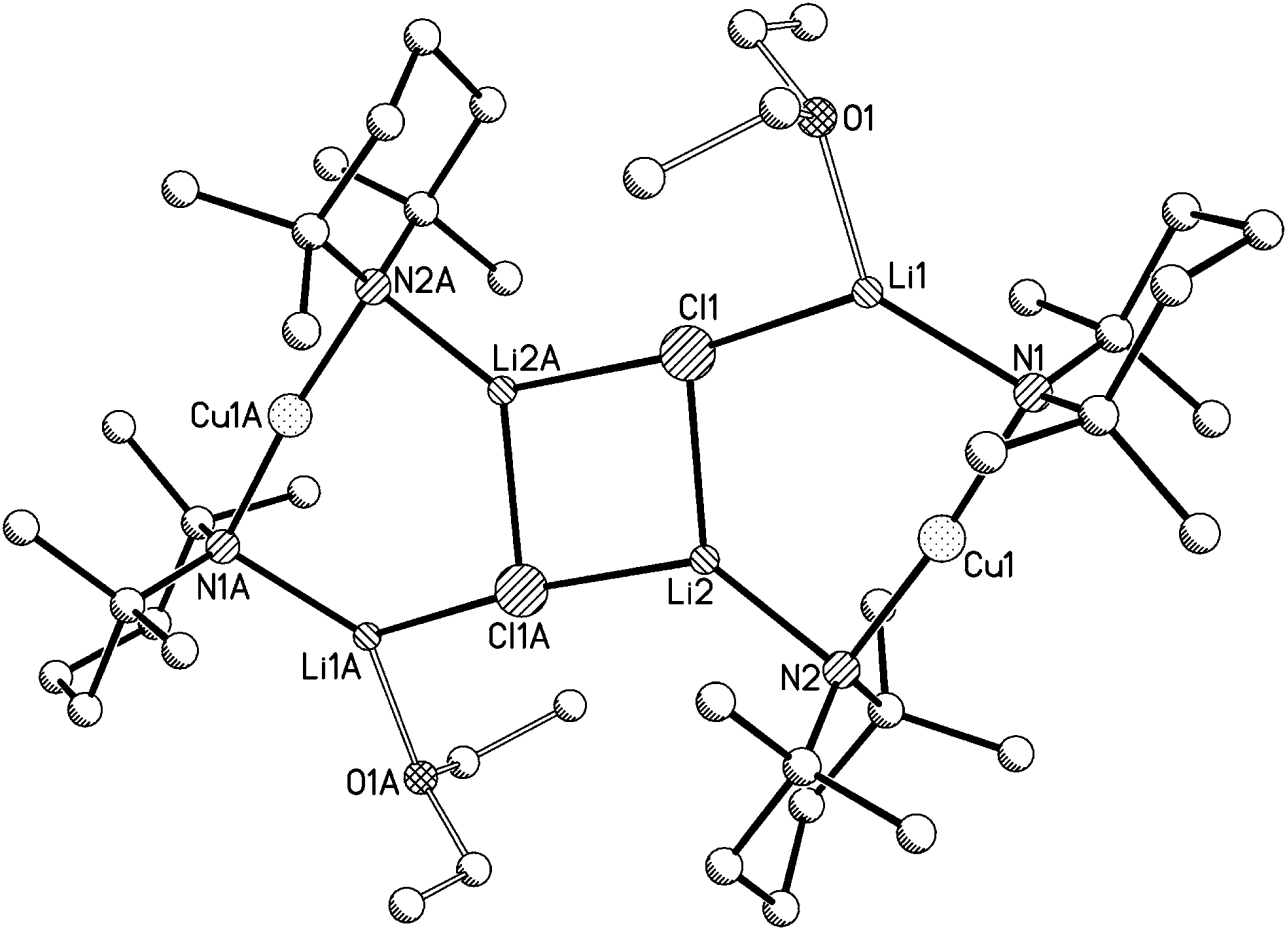
[{(TMP)_2_Cu(Cl)Li_2_(OEt_2_)}_2_] (2). H atoms are omitted. Selected bond lengths [Å] and angles [°]: Li1-Cl1 2.344(6), Li2-Cl1 2.332(7), Li1-N1 2.024(6), Li2-N2 1.953(7), Li1-N1-Cu1 90.4(2), Li2-N2-Cu1 91.8(2), Cl1-Li1-N1 125.2(3), Cl1-Li2-N2 127.3(3).

Recent studies have suggested the importance of steric effects in controlling amidocuprate reactivity.[3] To further probe this issue we have investigated the effect of replacing TMP with less bulky DMP (*cis*-2,6-dimethylpiperidide). Notably, DMPH also retails at a fraction of the cost of TMPH.[14] In the present case, DMPLi was added to CuX in the presence of Lewis base (bulk or 1 or 2 equiv Et_2_O with respect to Cu). Attempts to isolate a product when X=CN are yet to bear fruit. However, for X=Hal a remarkable series of structurally analogous complexes was obtained (Scheme 2 and illustrated for X=Cl in Figure 3).

**Scheme 2:**
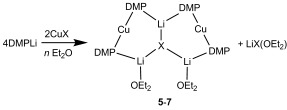
Synthesis of 5 (M=Cu, X=Cl, *n*=bulk), 6 (M=Cu, X=Br; *n*=bulk or 2 equiv with respect to Cu) and 7 (M=Cu, X=I; *n*=1 equiv with respect to Cu).

**Figure 3:**
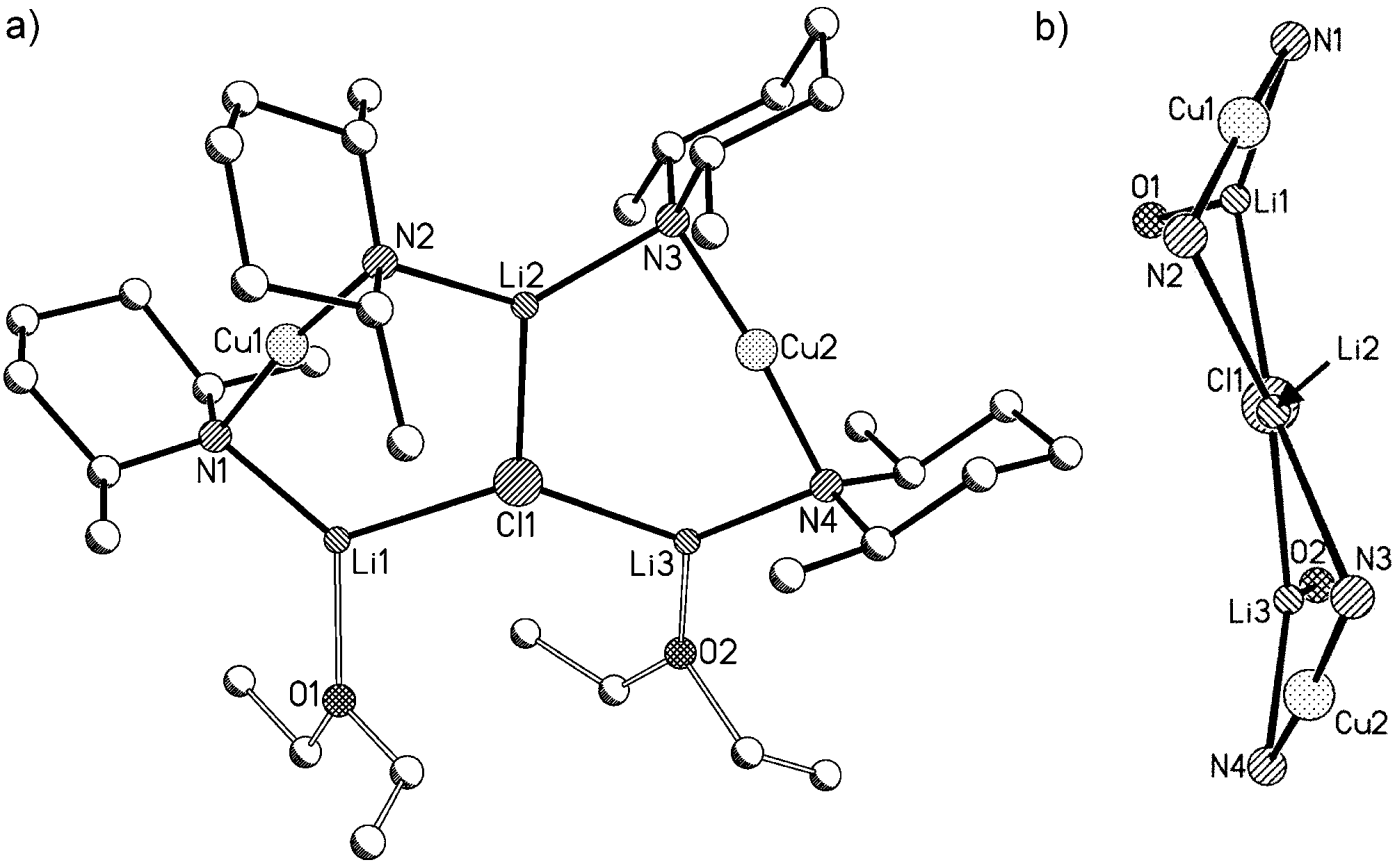
Adduct [{(DMP)_2_CuLi(OEt_2_)}_2_LiCl] (5). H atoms and minor Et_2_O disorder are omitted and the adduct core is viewed along the Li2=Cl1 axis in (b). Selected bond lengths [Å] and angles [°]: Li1-Cl1 2.354(6), Li2-Cl1 2.412(7), Li3-Cl1 2.301(7), Li1-N1 1.986(8), Li2-N2 2.035(7), Li2-N3 2.054(6), Li3-N4 1.970(9), Li1-N1-Cu1 87.6(2), Li2-N2-Cu1 94.1(2), Li2-N3-Cu2 94.2(2), Li3-N4-Cu2 90.1(2), N2-Li2-N3 129.2(4), Li1-Cl1-Li3 139.5(2).

For the use of X=Cl, the presence of bulk Et_2_O (Scheme 2) allowed the isolation of crystals that ^1^H NMR spectroscopy suggested contained Et_2_O and DMP in a 1:2 ratio. However, the observable presence of an NH resonance (at *δ*=0.85 ppm) was inconsistent with the spectral data obtained for **1**–**4**. Crystallographic analysis revealed a unique triangulated structure based on a lithium halide core and having the formulation [{(DMP)_2_CuLi(OEt_2_)}_2_LiCl] (**5**). This identification suggests that the (reproducible) observation of DMPH in solution is attributable to extreme moisture sensitivity in spite of the storage of deuterated solvents over a fresh Na mirror. The solid-state structure of **5** can be viewed as representing the first full characterisation of an adduct between a Lipshutz- and a Gilman-type cuprate.

The formation of this new class of cuprate adduct was next replicated using CuBr in the presence of Et_2_O in order to prepare [{(DMP)_2_CuLi(OEt_2_)}_2_LiBr] (**6**). Initial attempts used *n*BuLi (1 equiv with respect to amine) in the preparation of DMPLi. In the case of bulk Et_2_O this led to **6** (Scheme 2).[15] In contrast, the use of 2 equiv Et_2_O afforded [{(DMP)_2_CuLi(OEt_2_)}_1.45_{(DMP)_2_CuLi(DMPH)}_0.55_LiBr] (**6′**). This problem could be overcome, and the reproducible formation of **6** was obtained, by using 1.1 equiv *n*BuLi.[15]

Whereas the preparations of **5** and **6** used solvent conditions of bulk Et_2_O (for Cl) or of either bulk or limited Et_2_O (for Br), attempts to prepare the iodide analogue required that strictly limited quantities of Et_2_O be present. The use of bulk donor failed to afford isolable material, whereas the presence of 2 equiv Et_2_O with respect to Cu afforded only LiI(OEt_2_).[16] However, the documented solubility of lithium iodide in Et_2_O led us to suspect that this was causing the salt to largely remain in solution during filtration of the reaction mixture and to be subsequently crystallising. The amount of donor solvent was therefore further restricted to promote lithium iodide precipitation and removal. Storage of the resulting filtrate at −27 °C yielded crystalline blocks that X-ray crystallography confirmed to be [{(DMP)_2_CuLi(OEt_2_)}_2_LiI] (**7**; Scheme 2).[15] As with **5** and **6**, the spectroscopic observation of NH was interpreted in terms of extreme moisture sensitivity.

The structures of **6** and **7** are highly analogous to that of **5** and all exhibit approximate *C*_2_ symmetry about a central lithium halide axis, as shown representatively in Figure 3. In each case the halide shows triangulated coordination and two types of bond to Li^+^, with Li2=X being relatively extended (Li2=X 2.412(7), 2.592(7) and 2.971(16) Å in **5**, **6** and **7**, respectively). The Li1/3=X bonds are somewhat inequivalent: 2.354(6)/2.301(7), 2.474(8)/2.515(8) and 2.720(13)/2.667(14) Å in **5**, **6** and **7**, respectively. As would be expected, the metal=halide bonds extend as Group VII is descended. However, this extension is not consistent and the ratio between Li2=X and the mean of Li1/3=X is greater for iodide (1.10) than for chloride or bromide (1.04 in either case). This suggests that, rather than simply attributing this bond extension to the ionic radius of the halide, competition between metal stabilisation by hard and soft donors must also be considered. Thus, in the presence of soft iodide, Li2 is more inclined to be stabilised by the N2/3-based DMP ligands. This is reflected also in the Li2-N-Cu angles, which increase in response to the higher halide: Li2-N2/3-Cu1/2 94.1(2)/94.2(2)° (**5**), 97.3(2)/97.5(3)° (**6**), 100.1(5)/101.2(5)° (**7**). A similar trend is seen for the remaining two (N1/4-based) ligands, though, consistent with the shorter Li1/3=X bonds, the angles are smaller: Li1/3-N1/4-Cu1/2 87.6(2)/90.1(2)° (**5**), 92.5(3)/89.0(3)° (**6**), 96.1(5)/96.8(5)° (**7**). The asymmetry in these angles at nitrogen contrasts with the more symmetrical rings in **2**–**4**, in which the difference between Cu-N-Li and Cu-N-Li(OEt_2_) was never more than 3°.

Lastly, treatment of DMPLi with CuBr in dry toluene followed by recrystallisation in the presence of THF at −27 °C yielded [{(DMP)_2_CuLi(thf)_2_}_2_LiBr] (**8**) and established that adduct formation is not limited to the deployment of Et_2_O (Figure 4). The two THF-solvated Li^+^ ions are now pseudotetrahedral. Although the crystal structure of **8** is largely analogous to that of **6**, the effect of using a stronger Lewis base can be noted. Whereas Li2=N2/3 bonds are essentially unaffected, both Li1=N1 and Li3=N4 are extended in **8**. Similarly, Li1/3=Br1 increases significantly from 2.474(8)/2.515(8) Å in **6** to 2.609(11)/2.602(12) Å in **8**. The asymmetry in the Li-N-Cu bond angles noted in **6** is also now absent; the four angles in **8** being essentially identical. Lastly, evidence for additional stability conferred by the four THF molecules in **8** comes from the observation of a substantially smaller NH resonance in the ^1^H NMR spectrum (c.f. **5**–**7**).[15]

**Figure 4:**
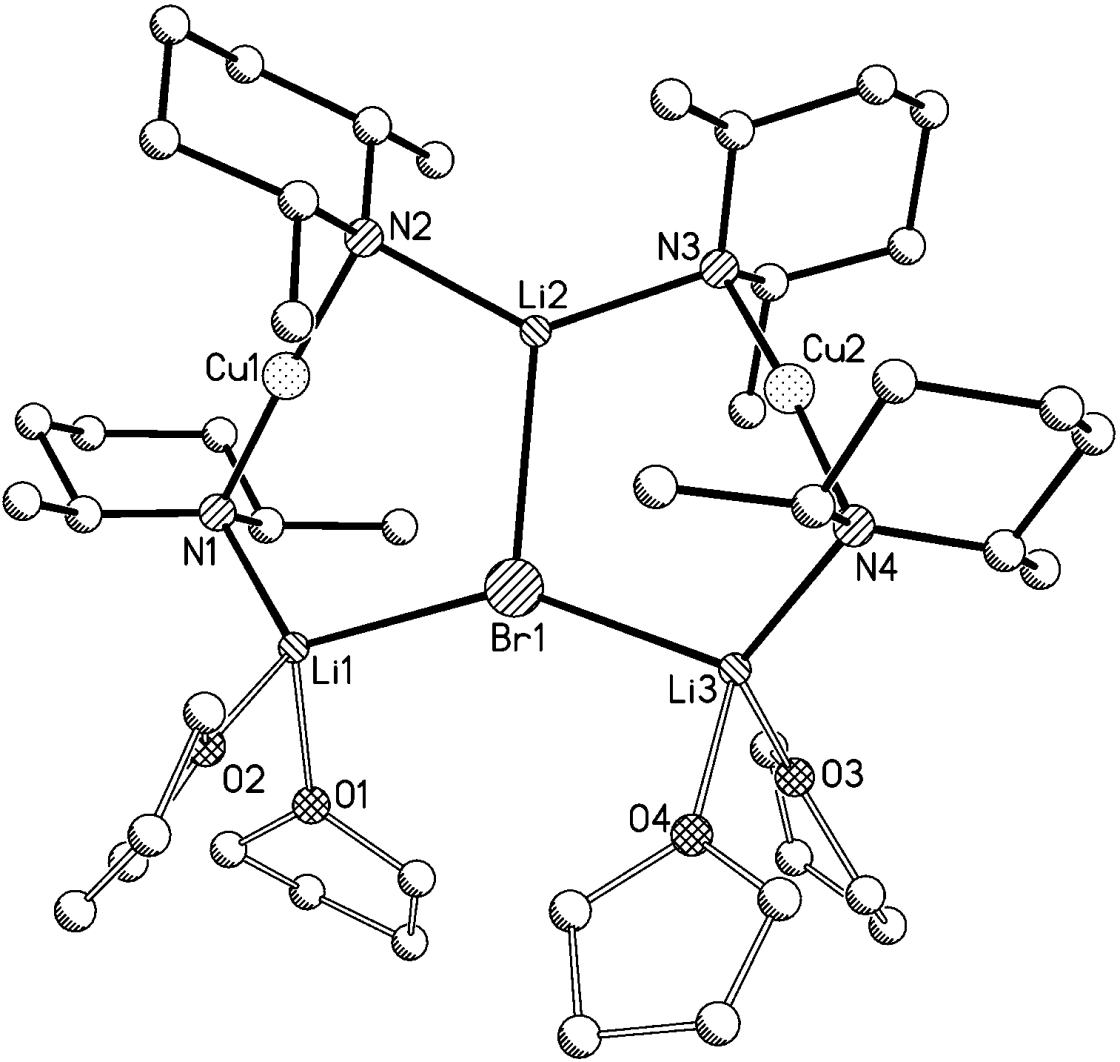
Adduct [{(DMP)_2_CuLi(thf)_2_}_2_LiBr] (8). H atoms are omitted. Selected bond lengths [Å] and angles [°]: Li1-Br1 2.609(11), Li2-Br1 2.677(11), Li3-Br1 2.602(12), Li1-N1 2.067(13), Li2-N2 2.045(11), Li2-N3 2.029(11), Li3-N4 2.094(15), Li1-N1-Cu1 94.3(4), Li2-N2-Cu1 93.9(4), Li2-N3-Cu2 94.2(4), Li3-N4-Cu2 92.0(4), N2-Li2-N3 132.2(6), Li1-Br1-Li3 147.3(4).

Although ^1^H NMR spectroscopy on adducts **5**–**7** suggests some sensitivity towards trace moisture, ^7^Li NMR spectroscopic analysis is consistent with a significant level of retention of the solid-state structures. In all cases a low-field signal (at *δ*=2.16–2.18 ppm) matches the dominant signal (at *δ*=2.20 ppm) in a DMPLi reference spectrum. For each of **5**, **6** and **7** in [D_6_]benzene the dominant signals are seen at *δ*=1.83–1.84 and 1.41–1.50 ppm in a 1:2 ratio, consistent with the crystallographic data. In the case of **6′**, the spectrum is more complicated, yet still consistent with crystallography. The presence of Li(DMPH) now introduces a signal at *δ*=1.66 ppm. However, the proximity of this to the *δ*=1.48 ppm signal attributable to Li(OEt_2_) prevents their separate integration. Lastly, for **8** in [D_6_]benzene a single environment is observed by ^7^Li NMR methods, and we attribute this to the four THF molecules present in **8**, which create a more polar medium than the two Et_2_O/DMPH molecules in **5**–**7**.

Subsequent investigation focused on the reasons for the transition in structure type from dimers **1**–**4** to adducts **5**–**8**. The possibility that solvent identity or quantity was a determining factor having been removed, competition was presumed to be dictated by the amide. This can be seen from the chloride species shown in Figures 2 and 3. The two TMP ligands associated with any given Cu atom (see N1, N2 in Figure 2) project away from one another so as to lie *endo*,*endo* with respect to the structure core (Figure 5, left). In contrast, the presence of DMP much reduces steric interaction between the methyl groups in the pair of amide ligands, allowing the piperidide rings to reside face-on to each other in a way that would be sterically precluded for TMP. The consequence of the face-on motif adopted by the DMP ligands is that they project away from the aggregate core in *exo*,*exo* fashion (Figure 5, right). This configuration of the DMP ligands in **5**–**8** also avoids steric congestion between the two amides that are bonded to the single unsolvated Li^+^ centre in the structure.

**Figure 5:**
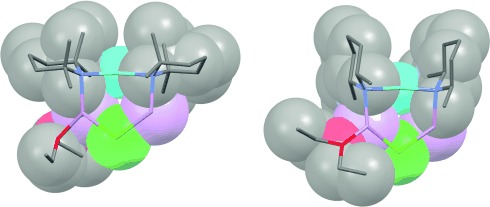
Structures of the Lipshutz-type monomers incorporated in TMP-based 2 (left) and DMP-based 5 (right) highlighting the *endo*,*endo* and *exo*,*exo* amide orientations.

We already know that Gilman-type cuprates show inferior DoC activity when compared to their Lipshutz-type analogues.[3] However, we have also established that DoC reactivity actually requires monomeric Gilman-type reagents *accessed from a Lipshutz-type precursor*,[6] and we here reinforce the importance of LiHal inclusion in amidocuprate chemistry (Scheme 3). In THF, *N*,*N*-diisopropylbenzamide reacted to give 2-iodo-*N*,*N*-diisopropylbenzamide (**9**) in 80 % yield using DMPLi, CuBr and the benzamide in a 4:2:1 ratio (i.e., 2 equiv Lipshutz-type Cu per arene) prior to I_2_ work-up. Meanwhile, dissolution of pre-isolated **6** and *N*,*N*-diisopropylbenzamide in a 1:1 ratio (i.e., 2 equiv Cu per arene) gave **9** in an essentially identical yield of 82 %. These data show that an adduct such as **6** can, like a Lipshutz-type dimer,[6] be viewed as an efficient source of reactive Gilman-type monomers.

**Scheme 3:**
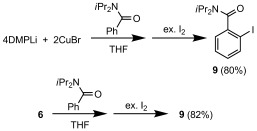
Directed *ortho* iodination using DMPLi, CuBr and *N*,*N*-diisopropylbenzamide in a 4:2:1 ratio (top) or 6 and *N*,*N*-diisopropylbenzamide in a 1:1 ratio (bottom).

We sought to probe the relationship between Lipshutz- and Gilman-type dimers using DFT methods (Scheme 4).[17] Results obtained with the simplified (Me_2_N)_2_CuLi(OMe_2_)+LiCl system[3], [6], [8] suggest that Lipshutz-type dimer **L_D_** exhibits an enthaplic preference (Δ*E*=−13.7 kcal mol^−1^) for eliminating a lithium halide solvate and forming a Gilman-type dimer, **G_D_**.[15] Meanwhile, a small entropy decrease, consistent with solvation of the eliminated halide, explains a slight increase (+4.5 kcal mol^−1^) in Δ*G*. Though the adduct between a Lipshutz- and a Gilman-type monomer (**L_M_G_M_**) is not the preferred cuprate of the three, the energetic balance between species is a fine one. Lastly, the ability of **L_M_G_M_** to associate with a reagent, such as *N*,*N*-dimethylbenzamide, prior to effecting a DoC reaction has been investigated.[15], [17] Results indicate that the conversion of **L_M_G_M_** to a complex between **G_M_** (Me_2_N)_2_CuLi(OMe_2_) and *N*,*N*-dimethylbenzamide along with 0.5 **L_D_** is accompanied by a change in Δ*G* of only +6.1 kcal mol^−1^.[15] This energy profile suggests a route to a *N*,*N*-dimethylbenzamide–**G_M_** adduct that compares favourably with that recently calculated using a Lipshutz monomer as the starting point,[6] and reinforces the view that **L_M_G_M_** adducts, such as **6**, represent viable DoC reagents.

**Scheme 4:**
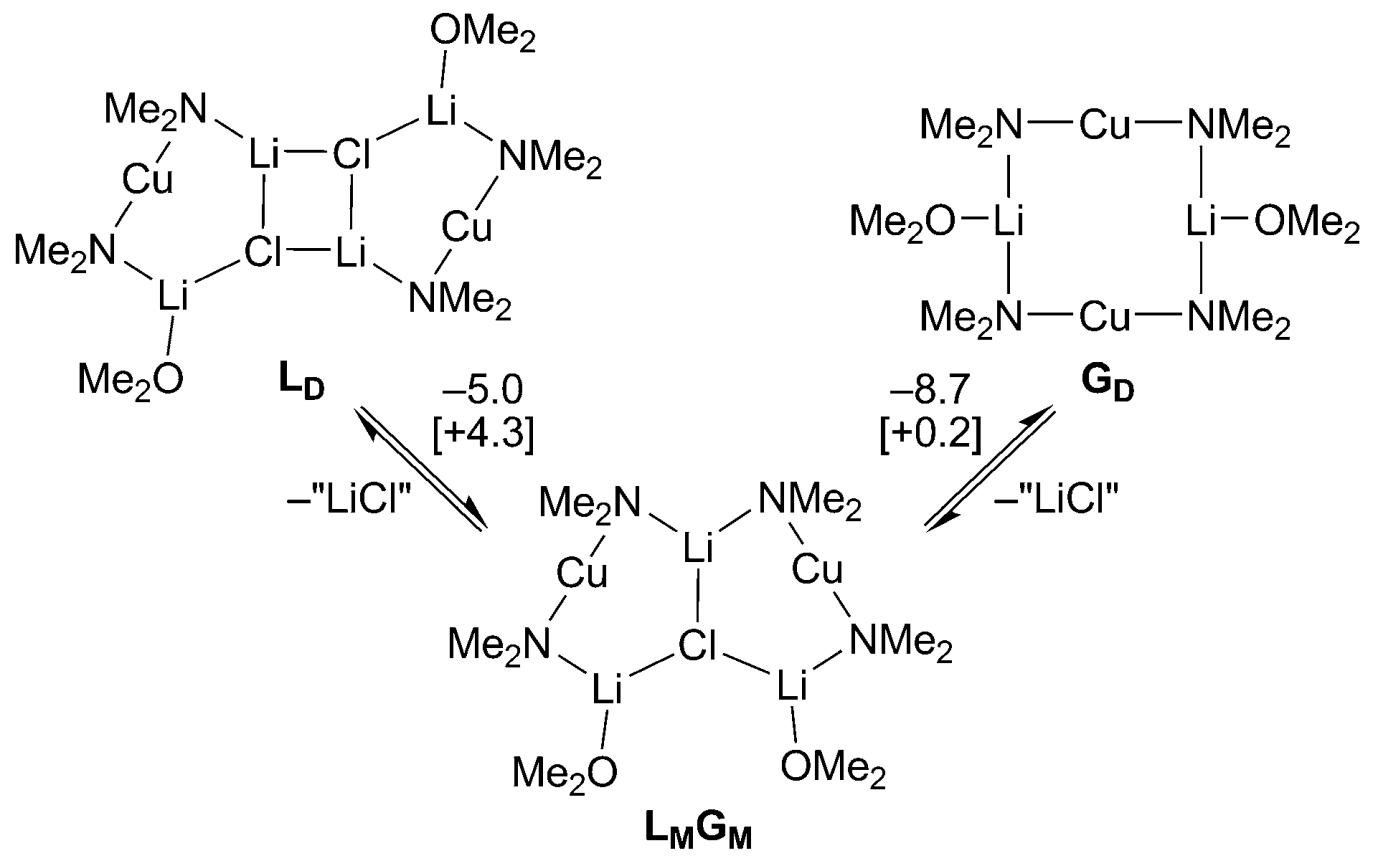
The interconversion of Lipshutz- and Gilman-type dimers via adduct [(Me_2_N)_2_CuLi(OMe_2_)]_2_LiCl (L_D_-L_M_G_M_-G_D_) at B3LYP/SVP level.[15], [17] Δ*E* [Δ*G*] are in kcal mol^−1^. “LiCl” is ^1^/_4_ [LiCl(OMe_2_)]_4_−OMe_2_.

To conclude, a previously unexplored class of triangulated lithium amidocuprate best viewed as a 1:1 adduct between Gilman- and Lipshutz-type monomers, is reported. The formation of **5**–**8** can be viewed as resulting from the abstraction of lithium halide from a Lipshutz-type dimer and the relative orientations of the amide ligands in both dimers **2**–**4** and adducts **5**–**8** can be rationalised sterically. Derivatisations of an aromatic tertiary amide undertaken with: 1) 2:1 DMPLi/CuBr, and 2) **6** reinforce the importance of LiX-containing systems in amidocuprate reactivity.[6] In both cases, high conversion is achieved using bases made with DMPH, suggesting major cost benefits.[14] To improve our mechanistic understanding, we have now initiated a detailed study of the solution behaviour of these adducts.[18] We are also seeking to use various amines to probe the relationship between ligand bulk and structure type.

CCDC-964430–964439 http://www.ccdc.cam.ac.uk/cgi-bin/catreq.cgicontains the supplementary crystallographic data for this paper. These data can be obtained free of charge from The Cambridge Crystallographic Data Centre via http://www.ccdc.cam.ac.uk/data_request/cif.
