# Supplementary material for: Structural Effects in Lithiocuprate Chemistry: The Elucidation of Reactive Pentametallic Complexes
Source: Chemistry. 2014 Feb 19;20(14):3908–12. doi: 10.1002/chem.201304824 (PMC4497349; doi:10.1002/chem.201304824)
Supplement: Supplementary file 1 [file chem0020-3908-sd1.pdf]

CHEMISTRY
A European Journal

Supporting Information

© Copyright Wiley-VCH Verlag GmbH & Co. KGaA, 69451 Weinheim, 2014

Structural Effects in Lithiocuprate Chemistry: The Elucidation of Reactive Pentametallic Complexes

Philip J. Harford,[a] Andrew J. Peel,[a] Joseph P. Taylor,[a] Shinsuke Komagawa,[b]
Paul R. Raithby,[c] Thomas P. Robinson,[c] Masanobu Uchiyama,*[b] and
Andrew E. H. Wheatley*[a]

chem_201304824_sm_miscellaneous_information.pdf

Table of Contents

1. X-ray Crystallographic Analysis

1-1. General synthetic and analytical details S-3

1-2. General crystallographic details S-3

1-3. Synthesis and characterization of **1** S-4

1-4. Synthesis and characterization of **2** S-5

1-5. Synthesis and characterization of **3** S-6

1-6. Synthesis and characterization of **4** S-7

1-7. Synthesis and characterization of **5** S-9

1-8. Synthesis and characterization of **6** S-10

1-9. Synthesis and characterization of **6´** S-12

1-10. Synthesis and characterization of **7** S-13

1-11. Synthesis and characterization of **8** S-15

2. Directed *ortho* Cupration Reactions

2-1. Preparation of 2-iodo-*N*,*N*-diisopropylbenzamide using a Lipshutz formulation base S-17

2-2. Preparation of 2-iodo-*N*,*N*-diisopropylbenzamide using pre-isolated **6** S-17

3. Computational Section

3-1. Details of computational methods S-18

3-2. Cartesian coordinates and energies S-19

4. References S-23

1. X-ray Crystallographic Analysis

1-1. General synthetic and analytical details.

All reactions and manipulations were carried out under an inert atmosphere of dry nitrogen, using standard double manifold and glove-box techniques. Solvents were distilled off sodium (toluene) or sodium-potassium amalgam (THF, Et_2O, hexane) immediately prior to use. Copper(I) reagents and the amines *cis*-2,6-dimethylpiperidine (DMPH) and 2,2,6,6-tetramethylpiperidine (TMPH) were purchased from Aldrich and *n*BuLi was purchased from Acros. The amines were stored over molecular sieves (4 Å) and all purchased chemicals were used without further purification. NMR data were collected on a Bruker DRX 400 (400.140 MHz for ^{1}H, 100.615 MHz for ^{13}C) or 500 (500.200 MHz for ^{1}H, 125.775 MHz for ^{13}C, 194.397 MHz for ^{7}Li) FT NMR spectrometer. Spectra were obtained at 298K and chemical shifts are internally referenced to the deuterated solvent and calculated relative to TMS except for ^{7}Li, for which an external reference was used (1 M LiOH in D_2O). Chemical shifts are expressed in δ ppm. The following abbreviations are used: s = singlet, d = doublet, m = multiplet, q = quartet. NMR solvents were stored over a freshly prepared Na mirror.

1-2. General Crystallographic Details.

Data were collected using the 'oil drop technique'[1] to mount crystals on either a Nonius Kappa-CCD or an Agilent Gemini A-Ultra diffractometer equipped with an Oxford Cryostream low-temperature device. Structures were solved using direct methods,[2] with refinement, based on F^2, by full-matrix least squares.[3] Non-hydrogen atoms were refined anisotropically and a riding model with idealised geometry was employed for the refinement of H-atoms (except for the NH in **6´**). For **4** the iodide centres were each refined over two positions with occupancy summing to unity. For **5** one methyl group in each coordinated ether was refined in separate positions with occupancy summing to unity. The associated methylene groups had EXYZ and EADP constraints applied to them, but the associated H-atoms were included to take up appropriate positions. For **6** a) all centres in one ether group were refined over two positions with occupancy summing to unity. For **6** b) the distances in the coordinated ether groups were fixed using the DFIX command. All oxygen and carbon atoms in the ether groups were modelled isotropically as the model was not good enough to support anisotropic refinement. The low value of _diffrn_measured_fraction_theta_full can be attributed to a weakly diffracting crystal, and there was no significant data at high 2 theta angles. For **6´** refinement of the main part of the structure $[(DMP)_2CuLi]_2 \cdot LiBr$ proved facile, however the solvent molecules coordinated to two of the lithium atoms proved less simple to model. The ether molecule coordinating to Li1 was refined in separate positions with the occupancy summing to unity. In addition, the distances were fixed using the DFIX command. Coordinating to Li3 there is a mixture of DMPH and ether groups, with three of the atomic sites, C29, C30 and C34, found to be overlapping. In these cases the atoms were split and the parts constrained using EXYZ and EADP. N5/O2 and C35/C35´ were refined over two different sites, settling at a ratio of 55 %: 45 % amine:ether. The distances in this ether group and the corresponding distances in the amine were fixed using the DFIX command. The amine proton was assigned to a *q* peak and subsequently fixed using the DFIX and DANG commands. The SIMU and DELU commands were used to restrain the parameters of C29, C29´, C35´ and C36´. The difference in the calculated and reported structures is due to rounding errors in the occupancies of the coordinated amine/ether. For **7** the iodide centres were each refined over two positions with occupancy summing to unity, and SIMU and

DELU commands were used to restrain the parameters of the carbon atoms in one of the ether groups. Data for all structures were collected with a redundancy of greater than 1 using an area detector, and in the data reduction an absorption correction was carried out by default using SORTAV.[4] Crystallographic data (excluding structure factors) have been deposited with the Cambridge Crystallographic Data Centre as supplementary publications CCDC-964430 – 964439. Copies of the data can be obtained free of charge on application to CCDC, 12 Union Road, Cambridge CB2 1EZ, UK (fax: +44 1223 336033; e-mail: deposit@ccdc.cam.ac.uk).

1-3. Synthesis and characterization of 1

*n*BuLi (1.25 mL, 1.6 M in hexanes, 2 mmol) was added dropwise to a solution of TMPH (0.34 mL, 2 mmol) in dry toluene (2 mL) under N_2 at –78 °C. The resulting solution of TMPLi was allowed to reach room temperature. It was then added to a suspension of copper(I) cyanide (90 mg, 1 mmol) in dry toluene (2 mL) under N_2 at –78 °C. The solvent was removed and dry Et_2O (8 mL) was added. The resulting solution was reduced to *ca*. 3 mL volume and 15 mL hexane was added, whereupon the mixture was filtered. Storage of the resulting yellow-white solution at –27 °C for 24 hours gave colourless blocks of **1**. Yield 190 mg (41 %) wrt CuCN, m. p. 191 ºC dec. X-ray crystal data: $C_{46}H_{92}Cu_2Li_4N_6O_2$, M = 916.10, monoclinic, space group $P2_1/c$, a = 8.1679(2), b = 26.2989(6) c = 13.2527 Å, β = 106.5320(10) °, V = 2729.09(12) Å^3, Z = 2, ρ_{calcd} = 1.115 g cm^{-3}, Mo-K$_\alpha$ radiation, λ = 0.71073 Å, μ = 0.816 mm^{-1}, T = 180(2)K. 19055 data (6349 unique, R_{int} = 0.1031, θ < 27.88 °) were collected. $wR2 = \{\Sigma[w(F_o^2-F_c^2)^2]/\Sigma[w(F_o^2)^2]\}^{1/2}$ = 0.3413, conventional R = 0.1241 on F values of 2997 reflections with $F^2 > 2\sigma(F^2)$, S = 1.055, 281 parameters. Residual electron density extrema ±2.526 eÅ^{-3}.

^{1}H NMR (500 MHz, 298K, d_8-THF): δ 3.42 (q, 4H, ^{3}J(H,H) = 7 Hz; Et_2O), 1.65 (br, m, 4H; TMP-2,6), 1.33 (br, m, 4H; TMP-4), 1.22 (br, m, 4H; TMP-3,5) 1.15 (t, 6H, ^{3}J(H,H) = 7 Hz; Et_2O), 1.12 (br, m, 18H, TMP-Me), 1.09 (m, 6H, TMP-Me)

^{13}C NMR (100 MHz, 298K, d_8-THF): δ 66.3 (Et_2O), 53.6 (TMP-2), 39.2 (TMP-3,5), 37.8 (TMP-Me), 32.2 (TMP-Me), 19.3 (TMP-4), 15.7 (Et_2O)

Elemental analysis calcd (%) for $C_{46}H_{92}Cu_2Li_4N_6O_2$: C 60.31, H 10.12, N 9.17; found: C 60.01, H 9.97, N 9.22

N(3)-Li(1)	1.991(15)	C(5)-N(3)	1.133(11)
N(3A)-Li(1)	2.134(14)	N(1)-Cu(1)-N(2)	178.9(2)
Cu(1)-N(1)	1.926(5)	N(3)–Li(1)–N(3A)	92.4(6)
Cu(1)-N(2)	1.909(5)	N(3A)–Li(1)–N(2)	137.7(7)
N(1)-Li(2)	2.014(18)	C(5A)–Li(2)–N(1)	122.6(7)
N(2)-Li(1)	2.009(13)	Li(1)–N(2)–Cu(1)	94.6(5)
C(5)-Li(2A)	2.089(17)	Li(2)–N(1)–Cu(1)	89.7(4)
C(5)··Li(1A)	2.572(15)		

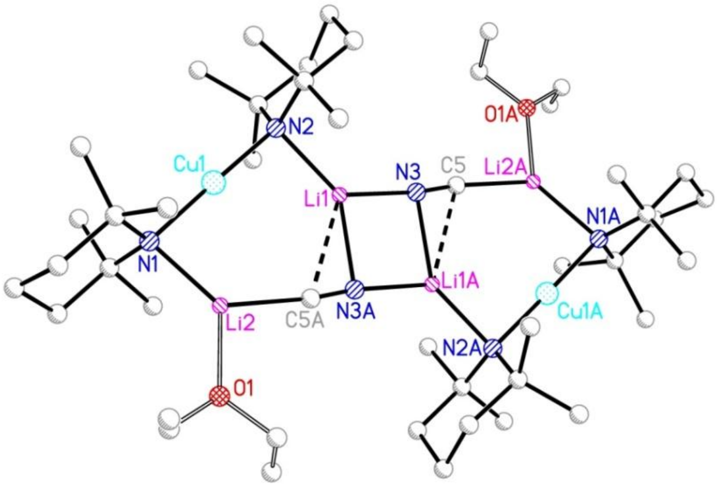

Figure S1. Lipshutz dimer $[\{(TMP)_2Cu(CN)Li_2(OEt_2)\}_2]$ **1**. H atoms omitted for clarity.

1-4. Synthesis and characterization of 2

A room temperature solution of TMPLi was prepared as for **1** and was added to a suspension of copper(I) chloride (99 mg, 1 mmol) in dry toluene (2 mL) under N_2 at –78 °C. The resulting mixture was allowed to reach room temperature. The solvent was removed and dry Et_2O (8 mL) was added, whereupon the mixture was filtered. The resulting solution was reduced to *ca*. 3 mL volume and 10 mL hexane was added. Storage of the resulting pale yellow solution at –27 °C for 72 hours gave colourless needles of **2**. Yield 60 mg (13 %) wrt CuCl, m. p. 190 °C dec. X-ray crystal data: $C_{44}H_{92}Cl_2Cu_2Li_4N_4O_2$, M = 934.96, monoclinic, space group $P2_1/n$, a = 8.1295(2), b = 21.4601(4), c = 15.3005(3) Å, β = 92.3120(10) °, V = 2667.15(10) Å^3, Z = 2, ρ_{calcd} = 1.164 g cm^{-3}, Mo-K$_\alpha$ radiation, λ = 0.71070 Å, μ = 0.932 mm^{-1}, T = 180(2)K. 30021 data (6088 unique, R_{int} = 0.0892, θ < 27.49 °) were collected. $wR2 = \{\Sigma[w(F_o^2-F_c^2)^2]/\Sigma[w(F_o^2)^2]\}^{1/2}$ = 0.1365, conventional R = 0.0582 on F values of 4397 reflections with $F^2 > 2\sigma(F^2)$, S = 1.062, 272 parameters. Residual electron density extrema ±0.908 eÅ^{-3}.

^{1}H NMR (500 MHz, 298K, [D$_6$]benzene): δ 3.46 (q, 4H, ^{3}J(H,H) = 7 Hz; Et_2O), 2.08 (br, m, 2H; TMP-3,5), 1.82 (br, m, 12H; TMP-Me), 1.71 – 1.67 (br, m, 12H; TMP-Me), 1.37 (br, m, 4H; TMP-4), 1.19 (br, m, 6H; TMP-3,5), 1.18 (t, 6H, ^{3}J(H,H) = 7 Hz; Et_2O)

^{13}C NMR (100 MHz, 298K, [D$_6$]benzene): δ 65.8 (Et_2O), 54.6 (TMP-2,6), 42.5 (TMP-3,5), 40.6 (TMP-Me), 35.0 (TMP-Me), 19.6 (TMP-4), 15.5 (Et_2O)

Elemental analysis calcd (%) for $C_{44}H_{92}Cl_2Cu_2Li_4N_4O_2$: C 56.52, H 9.92, N 5.99, Cl 7.48; found: C 55.71, H 9.68, N 6.95, Cl 7.96

Cl(1)-Li(1)	2.344(6)	N(2)-Li(2)	1.953(7)
Cl(1)-Li(2)	2.332(7)	Cl(1)–Li(2)–Cl(2)	100.5(3)
Cl(1)-Li(2A)	2.330(6)	Cl(1)–Li(1)–N(1)	125.2(3)
Cu(1)-N(1)	1.925(3)	Cl(1)–Li(2)–N(2)	127.3(3)
Cu(1)-N(2)	1.923(2)	Li(1)–N(1)–Cu(1)	90.4(2)
N(1)-Li(1)	2.024(6)	Li(2)–N(2)–Cu(1)	91.8(2)

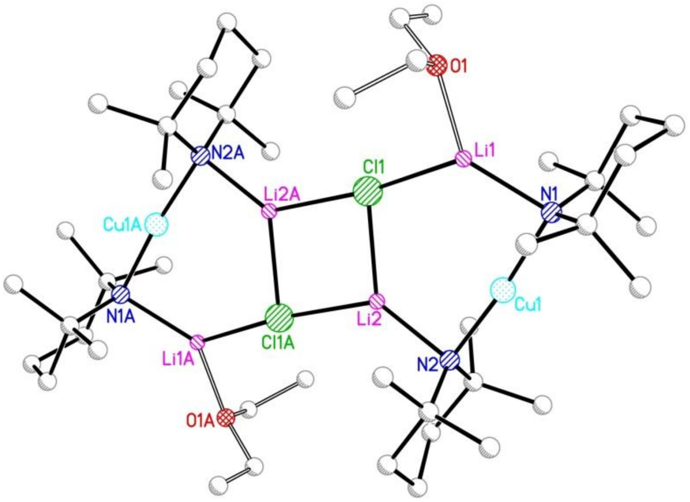

Figure S2. Lipshutz-type dimer [{(TMP)$_2$Cu(Cl)Li$_2$(OEt$_2$)}$_2$] **2**. H atoms omitted for clarity.

1-5. Synthesis and characterization of 3

a) A room temperature solution of TMPLi was prepared as for **1** and was added to a suspension of copper(I) bromide (143 mg, 1 mmol) in dry toluene (1 mL) under N_2 at –78 °C. The resulting mixture was allowed to reach room temperature. The solvent was removed and dry Et_2O (8 mL) was added, whereupon the mixture was filtered. The resulting solution was reduced to *ca*. 3 mL volume and 12 mL hexane was added, whereupon the mixture was filtered. Storage of the resulting yellow solution at –27 °C for 48 hours gave colourless blocks of **3**. Yield 91 mg (18 %) wrt CuBr, m. p. 193 ºC dec. X-ray crystal data: $C_{44}H_{92}Br_2Cu_2Li_4N_4O_2$, M = 1023.88, triclinic, space group P–1, a = 12.7782(2), b = 15.0503(2), c = 15.7127(2) Å, α = 86.357(5), β = 69.795(5), γ = 69.874(5) °, V = 2657.44(7) Å^3, Z = 2, ρ_{calcd} = 1.280 g cm^{-3}, Mo-K$_\alpha$ radiation, λ = 0.71073 Å, μ = 2.339 mm^{-1}, T = 180(2)K. 46172 data (12143 unique, R_{int} = 0.0601, θ < 27.47 °) were collected. $wR2 = \{\Sigma[w(F_o^2-F_c^2)^2]/\Sigma[w(F_o^2)^2]\}^{1/2}$ = 0.1573, conventional R = 0.0609 on F values of 9074 reflections with $F^2 > 2\sigma(F^2)$, S = 1.062, 543 parameters. Residual electron density extrema ±1.205 eÅ^{-3}.

^{1}H NMR (500 MHz, 298K, d$_8$-THF): δ 3.42 (q, 4H, ^{3}J(H,H) = 7 Hz; Et_2O), 1.71 – 1.62 (br, m, 12H; TMP-3,4,5), 1.32 (br, m, 12H; TMP-Me), 1.14 (t, 6H, ^{3}J(H,H) = 7 Hz; Et_2O), 1.08 (s, 12H; TMP-Me)

^{13}C NMR (125 MHz, 298K, d$_8$-THF): δ 66.3 (Et_2O), 50.1, 50.0 (TMP-2,6), 39.2 (TMP-3,5), 32.2 (TMP-Me), 31.2 (TMP-Me), 19.3 (TMP-4), 15.7 (Et_2O)

Elemental analysis calcd (%) for $C_{44}H_{92}Br_2Cu_2Li_4N_4O_2$: C 51.61, H 9.06, N 5.47, Br 15.61; found: C 50.77, H 8.74, N 5.47, Br 15.49

b) *n*BuLi (2.5 mL, 1.6 M in hexanes, 4 mmol) was added dropwise to a solution of TMPH (0.68 mL, 4 mmol) in dry toluene (3 mL) and dry Et_2O (0.42 mL) under N_2 at –78 °C. The resulting solution of TMPLi was allowed to reach room temperature. It was then added to a suspension of copper(I) bromide (286 mg, 2 mmol) in dry toluene (2 mL) under N_2 at –78 °C. The mixture was allowed to reach room temperature whereupon it was filtered. Storage at –27 °C for 36 hours gave colourless blocks of **3**. Yield 242 mg (24 %) wrt CuBr, m. p. 194 ºC dec. A crystallographic cell check verified the crystal data obtained by route a.

Br(1)-Li(1)	2.485(9)	N(3)-Li(3)	1.972(8)
Br(1)-Li(2)	2.556(8)	N(4)-Li(4)	1.994(9)
Br(1)-Li(3)	2.543(8)	Br(1)–Li(2)–Br(2)	99.6(3)
Br(2)-Li(1)	2.498(9)	Br(1)–Li(3)–Br(2)	98.1(3)
Br(2)-Li(3)	2.498(8)	Br(1)–Li(1)–N(1)	130.6(4)
Br(2)-Li(4)	2.546(8)	Br(1)–Li(2)–N(2)	126.9(4)
Cu(1)-N(1)	1.920(4)	Br(2)–Li(3)–N(3)	127.4(4)
Cu(1)-N(2)	1.914(4)	Br(2)–Li(4)–N(4)	124.7(4)
Cu(2)-N(3)	1.927(4)	Li(1)–N(1)–Cu(1)	92.1(3)
Cu(2)-N(4)	1.926(4)	Li(2)–N(2)–Cu(1)	89.1(3)
N(1)-Li(1)	1.976(9)	Li(3)–N(3)–Cu(2)	92.3(3)
N(2)-Li(2)	2.010(9)	Li(4)–N(4)–Cu(2)	88.4(3)

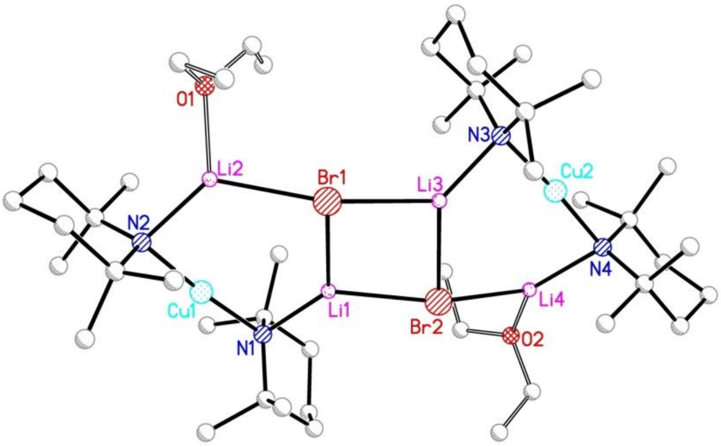

Figure S3. Lipshutz-type dimer [{$(TMP)_2Cu(Br)Li_2(OEt_2)$}$_2$] **3**. H atoms omitted for clarity.

1-6. Synthesis and characterization of 4

a) *n*BuLi (2.5 mL, 1.6 M in hexanes, 4 mmol) was added dropwise to a solution of TMPH (0.68 ml, 4 mmol) in dry toluene (2 mL) under N_2 at –78 °C. The resulting solution of TMPLi was allowed to reach room temperature. It was then added to a suspension of copper(I) iodide (380 mg, 2 mmol) in dry toluene (3 mL) under N_2 at –78 °C and the resulting mixture was allowed to reach room temperature. The solvent was removed and dry Et_2O (8 mL) was added, whereupon the mixture was filtered. The resulting solution was reduced to *ca*. 3 mL volume and 12 mL hexane was added. Storage at +5 °C for 24 hours gave yellow blocks of **4**. Yield 303 mg (27 %) wrt CuI, m. p. 185 °C dec. X-ray crystal data: $C_{44}H_{92}I_2Cu_2Li_4N_4O_2$, M = 1117.86, triclinic, space group P–1, a = 12.87700(10) , b = 15.0455(2), c = 16.1130(2) Å, α = 87.0190(10), β = 70.4260(10), γ = 70.2500(10) °, V = 2761.72(5) Å^3, Z = 2, ρ_{calcd} = 1.344 g cm^{-3}, Mo-K$_\alpha$ radiation, λ = 0.71073 Å, μ = 1.922 mm^{-1}, T = 180(2)K. 38687 data (15904 unique, R_{int} = 0.0412, θ < 30.03 °) were collected. $wR2 = \{\Sigma[w(F_o^2-F_c^2)^2]/\Sigma[w(F_o^2)^2]\}^{1/2}$ = 0.0859, conventional R = 0.0300 on F values of 12468 reflections with $F^2 > 2\sigma(F^2)$, S = 1.036, 561 parameters. Residual electron density extrema ±0.651 eÅ^{-3}.

^1H NMR (500 MHz, 298K, [D$_6$]benzene): δ 3.45 (q, 4H, ^3J(H,H) = 7 Hz; Et$_2$O), 1.90 (br, m, 2H; TMP-3,5), 1.84 – 1.51 (m, 24H; TMP-Me), 1.31 (m, 4H; TMP-4), 1.23 (br, m, 6H; TMP-3,5), 1.23 (t, 6H, ^3J(H,H) = 7 Hz; Et$_2$O)

^{13}C NMR (100 MHz, 298K, [D$_6$]benzene): δ 65.7 (Et$_2$O), 54.3 (TMP-2,6), 41.0 (TMP-3,5), 39.2 (TMP-Me), 34.3 (TMP-Me), 19.7 (TMP-4), 15.1 (Et$_2$O)

Elemental analysis calcd (%) for $C_{44}H_{92}I_2Cu_2Li_4N_4O_2$: C 47.27, H 8.30, N 5.01, I 22.70; found: C 46.53, H 8.23, N 5.11, I 22.80

b) *n*BuLi (2.5 mL, 1.6 M in hexanes, 4 mmol) was added dropwise to a solution of 2,2,6,6-tetramethylpiperidine (0.68 ml, 4 mmol) in dry toluene (3 mL) and dry ether (0.42 mL) under N_2 at –78 °C. The resulting solution of lithium 2,2,6,6-tetramethylpiperidide was allowed to reach room temperature. It was then added to a suspension of copper(I) iodide (380 mg, 2 mmol) in dry toluene (3 mL) under N_2 at –78 °C and the resulting mixture was allowed to reach room temperature. The mixture was filtered and storage of the resulting yellow solution at –27 °C for 36 h gave colourless blocks of **4**. Yield 188 mg (17 %) wrt CuI, m. p. 185 ºC dec. A crystallographic cell check verified the crystal data obtained by route a.

I(1)-Li(1)	2.747(4)	N(3)-Li(3)	1.966(4)
I(1)-Li(3)	2.730(4)	N(4)-Li(4)	1.994(3)
I(1)-Li(4)	2.743(4)	I(1)–Li(2)–I(2)	96.74(15)
I(2)-Li(1)	2.715(5)	I(1)–Li(3)–I(2)	95.71(14)
I(2)-Li(2)	2.866(5)	I(1)–Li(3)–N(3)	130.52(17)
I(2)-Li(3)	2.776(5)	I(1)–Li(4)–N(4)	123.68(16)
Cu(1)-N(1)	1.9191(15)	I(2)–Li(1)–N(1)	134.07(18)
Cu(1)-N(2)	1.9193(14)	I(2)–Li(2)–N(2)	125.53(18)
Cu(2)-N(3)	1.9264(15)	Li(1)–N(1)–Cu(1)	92.26(12)
Cu(2)-N(4)	1.9272(15)	Li(2)–N(2)–Cu(1)	89.93(11)
N(1)-Li(1)	1.964(4)	Li(3)–N(3)–Cu(2)	93.18(12)
N(2)-Li(2)	1.998(4)	Li(4)–N(4)–Cu(2)	89.62(11)

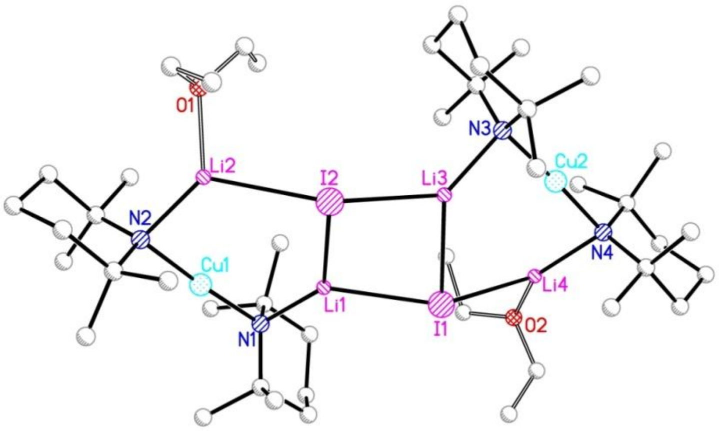

Figure S4. Lipshutz-type dimer [{(TMP)$_2$Cu(I)Li$_2$(OEt$_2$)}$_2$] **4**. H atoms and minor iodide disorder omitted for clarity.

1-7. Synthesis and characterization of 5

*n*BuLi (1.25 mL, 1.6 M in hexanes, 2 mmol) was added dropwise to a solution of DMPH (0.27 mL, 2 mmol) in dry Et_2O (2 mL) under N_2 at –78 °C. The resulting solution of DMPLi was allowed to reach room temperature. It was then added to a suspension of copper(I) chloride (99 mg, 1 mmol) in dry Et_2O (2 mL) under N_2 at –78 °C. The mixture was allowed to reach room temperature whereupon it was filtered and further dry Et_2O (1 mL) was added. Storage at –27 °C for 12 hours gave colourless blocks of **5**. Yield 46 mg (12 %), m. p. 121 ºC dec. X-ray crystal data: $C_{36}H_{76}ClCu_2Li_3N_4O_2$, M = 780.36, orthorhombic, space group $Pna2_1$, a = 14.877(5), b = 23.510(5), c = 12.600(5) Å, V = 4407(2) Å^3, Z = 4, ρ_{calcd} = 1.176 g cm^{-3}, Mo-K_α radiation, λ = 0.71073 Å, μ = 1.057 mm^{-1}, T = 173.15 K. 90225 data (13112 unique, R_{int} = 0.0567, θ < 31.029 °) were collected. $wR2 = \{\Sigma[w(F_o^2-F_c^2)^2]/\Sigma[w(F_o^2)^2]\}^{1/2}$ = 0.1429, conventional R = 0.0551 on F values of 11345 reflections with $F^2 > 2\sigma(F^2)$, S = 1.111, 457 parameters. Residual electron density extrema ±1.574 eÅ^{-3}.

^{1}H NMR (500 MHz, 298K, [D$_6$]benzene): δ 3.38 (q, 8H, ^3J(H,H) = 7 Hz; Et_2O), 3.08 – 2.55 (br, m, 8H; DMP-2,6), 1.88 – 1.58 (br, m, 24H; DMP-3,4,5), 1.52 (m, 20H; DMP-Me), 1.22 (t, 12H, ^3J(H,H) = 7 Hz; Et_2O), 1.07 (d, 4H; DMPH-Me), 0.85 (s, br, 1.5H; DMPH-NH)

^{13}C NMR (100 MHz, 298K, [D$_6$]benzene): δ 65.6 (Et_2O), 58.7 (DMP-2,6), 52.4 (DMPH-2,6), 39.4, 38.8 (DMP-3,5), 34.3 (DMPH-3,5), 28.5 (DMP-Me), 28.1, 27.3 (DMP-4), 25.4 (DMPH-4), 23.1 (DMPH-Me), 15.3 (Et_2O)

^{7}Li NMR (194 MHz, 298K, [D$_6$]benzene): δ 2.16 (br, s, 0.4Li; DMPLi), 1.83 (br, s, 1Li; **5**), 1.48 (br, s, 2Li·OEt_2; **5**), –0.51 (br, s, 0.2Li; unidentified)

Elemental analysis calcd (%) for $C_{36}H_{76}ClCu_2Li_3N_4O_2$: C 55.41, H 9.82, N 7.18, Cl 4.54; found: C 54.64, H 9.66, N 7.74, Cl 4.05

Cl(1)-Li(1)	2.354(6)	N(1)-Cu(1)-N(2)	172.42(12)
Cl(1)-Li(2)	2.412(7)	N(3)-Cu(2)-N(4)	174.43(14)
Cl(1)-Li(3)	2.301(7)	N(2)-Li(2)-N(3)	129.2(4)
Cu(1)-N(1)	1.905(3)	Li(1)–Cl(1)–Li(2)	109.1(2)
Cu(1)-N(2)	1.902(3)	Li(3)–Cl(1)–Li(2)	111.3(2)
Cu(2)-N(3)	1.891(3)	Cl(1)–Li(1)–N(1)	121.9(3)
Cu(2)-N(4)	1.914(3)	Cl(1)–Li(3)–N(4)	124.5(4)
N(1)-Li(1)	1.986(8)	Li(1)–N(1)–Cu(1)	87.6(2)
N(2)-Li(2)	2.035(7)	Li(2)–N(2)–Cu(1)	94.1(2)
N(3)-Li(2)	2.054(6)	Li(2)–N(3)–Cu(2)	94.2(2)
N(4)-Li(3)	1.970(9)	Li(3)–N(4)–Cu(2)	90.1(2)

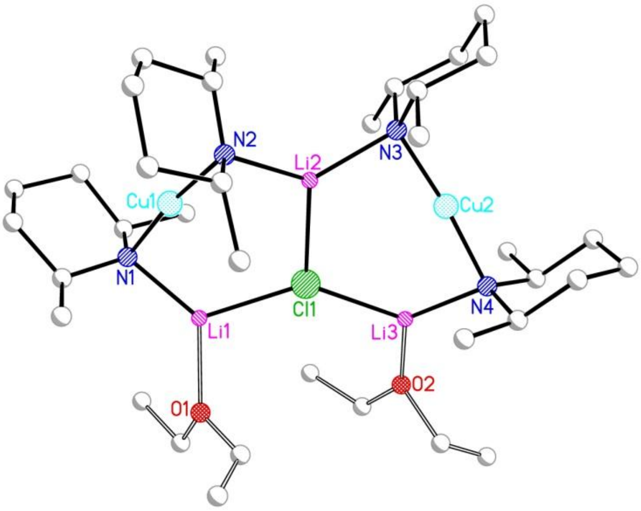

Figure S5. Adduct $[\{(DMP)_2CuLi(OEt_2)\}_2LiCl]$ **5**. H atoms and minor ether disorder omitted for clarity.

1-8. Synthesis and characterization of 6

a) A room temperature solution of DMPLi was prepared as for **5** and was added to a suspension of copper(I) bromide (143 mg, 1 mmol) in dry Et_2O (2 mL) under N_2 at –78 °C. The mixture was allowed to reach room temperature whereupon it was filtered and the resulting pale orange solution stored at –27 °C for 12 hours gave yellow blocks of **6**. Yield 88 mg (21 %) wrt CuBr, m. p. 95 ºC dec. X-ray crystal data: $C_{36}H_{76}BrCu_2Li_3N_4O_2$, M = 824.82, orthorhombic, space group $Pna2_1$, a = 14.8880(2), b = 23.7813(5), c = 12.6698(2) Å, V = 4485.82(13) Å^3, Z = 4, ρ_{calcd} = 1.221 g cm^{-3}, Mo-K$_\alpha$ radiation, λ = 0.71073 Å, μ = 1.870 mm^{-1}, T = 180(2) K. 16869 data (8233 unique, R_{int} = 0.0329, θ < 27.47 °) were collected. $wR2 = \{\Sigma[w(F_o^2-F_c^2)^2]/\Sigma[w(F_o^2)^2]\}^{1/2}$ = 0.0950, conventional R = 0.0420 on F values of 6987 reflections with $F^2 > 2\sigma(F^2)$, S = 1.015, 480 parameters. Residual electron density extrema ±0.661 eÅ^{-3}.

^{1}H NMR (500 MHz, 298K, [D$_6$]benzene): δ 3.38 (q, 8H, ^3J(H,H) = 7 Hz; Et_2O), 3.08 – 2.76 (br, m, 8H; DMP-2,6), 1.92 – 1.60 (br, m, 24H; DMP-3,4,5), 1.52 (m, 24H; DMP-Me), 1.21 (t, 12H, ^3J(H,H) = 7 Hz; Et_2O), 1.09 (br, s, 2H; DMP-Me), 0.85 (s, br, 1.3H; DMPH-NH)

^{13}C NMR (100 MHz, 298K, [D$_6$]benzene): δ 65.7 (Et_2O), 59.4 (DMP-2,6), 52.5 (DMPH-2,6), 40.9, 40.2, 38.8 (DMP-3,5), 34.1 (DMPH-3,5), 28.7, 28.0 (DMP-Me), 27.3 (DMP-4), 26.5 (DMPH-4), 23.0 (DMPH-Me), 15.2 (Et_2O)

^{7}Li NMR (194 MHz, 298K, [D$_6$]benzene): δ 2.18 (br, s, 0.4Li; TMPLi), 1.84 (br, s, 1Li; **6**), 1.48 (br, s, 2Li·OEt_2; **6**)

Elemental analysis calcd (%) for $C_{36}H_{76}BrCu_2Li_3N_4O_2$: C 52.42, H 9.29, N 6.79, Br 9.69; found: C 50.43, H 8.93, N 6.56, Br 10.25

b) *n*BuLi (4.14 mL, 1.6 M in hexanes, 6.6 mmol) was added dropwise to a solution of DMPH (0.81 mL, 6 mmol) in dry hexane (6 mL) and dry Et_2O (0.63 mL) under N_2 at –78 °C. The resulting solution of DMPLi was allowed to reach room temperature. It was then added to a suspension of copper(I) bromide (429 mg, 3 mmol) in dry hexane (3 mL) under N_2 at –78 °C. The mixture was allowed to reach room temperature, and then filtered and the resulting orange solution was stored at –27 °C yielding **6** as orange/brown blocks after

72 hours. Yield 584 mg, 47 % wrt CuBr, m. p. 95 ºC, dec. X-ray crystal data: $C_{36}H_{76}BrCu_2Li_3N_4O_2$, M = 824.82, triclinic, space group P–1, a = 12.4266(4), b = 12.7166(4), c = 16.5053(7) Å, α = 102.634(2), β = 95.762(2), γ = 113.775(2) °, V = 2276.57(14) Å^3, Z = 2, ρ_{calcd} = 1.203 g cm^{-3}, Mo-K$_\alpha$ radiation, λ = 0.71073 Å, μ = 1.842 mm^{-1}, T = 180(2) K. 12561 data (6862 unique, R_{int} = 0.0388, θ < 25.24 °) were collected. $wR2 = \{\Sigma[w(F_o^2-F_c^2)^2]/\Sigma[w(F_o^2)^2]\}^{1/2}$ = 0.2631, conventional R = 0.0842 on F values of 3859 reflections with $F^2 > 2\sigma(F^2)$, S = 1.087, 387 parameters. Residual electron density extrema ±1.242 eÅ^{-3}.

^{1}H NMR (500 MHz, 298K, [D$_6$]benzene): δ 3.35 (q, 8H, ^{3}J(H,H) = 7 Hz; Et_2O), 3.28 – 2.66 (m, 8H; DMP-2,6), 2.20 (br, m, 4H; DMP-4), 2.02 – 1.70 (m, 10H; DMP-3, 5), 1.62 (br, m, 10H DMP-3, 4, 5) 1.50 (d, 22H; DMP-Me), 1.12 (t, 12H, ^{3}J(H,H) = 7 Hz; Et_2O), 1.08 (d, 2H, DMP-Me), 0.85 (s, br, 0.6H; DMPH-NH)

^{13}C NMR (100MHz, 298K, [D$_6$]benzene): δ 65.7 (Et_2O), 58.6, 57.4 (DMP-2,6), 52.7 (DMPH-2,6) 39.6, 38.5 (DMP-3,5), 33.7 (DMPH-3,5), 27.4 (DMP-Me), 26.8 (DMP-4), 24.5 (DMPH-4), 22.7 (DMPH-Me), 14.7 (Et_2O)

^{7}Li NMR (194MHz, 298K, [D$_6$]benzene): δ 2.15 (br, s, 0.6Li; TMPLi), 1.83 (br, s, 1Li; **6**), 1.50 (br, s, 2Li·OEt_2; **6**)

Elemental analysis calcd (%) for $C_{36}H_{76}BrCu_2Li_3N_4O_2$: C 52.42, H 9.29, N 6.79, Br 9.69; found: C 52.32, H 9.16, N 7.75, Br 10.62

Br(1)-Li(1)	2.474(8)	N(1)-Cu(1)-N(2)	175.37(16)
Br(1)-Li(2)	2.592(7)	N(3)-Cu(2)-N(4)	173.98(14)
Br(1)-Li(3)	2.515(8)	N(2)-Li(2)-N(3)	133.0(4)
Cu(1)-N(1)	1.916(3)	Li(1)–Br(1)–Li(2)	108.9(2)
Cu(1)-N(2)	1.887(3)	Li(3)–Br(1)–Li(2)	106.5(2)
Cu(2)-N(3)	1.892(3)	Br(1)–Li(1)–N(1)	122.8(4)
Cu(2)-N(4)	1.912(3)	Br(1)–Li(3)–N(4)	120.3(4)
N(1)-Li(1)	1.955(9)	Li(1)–N(1)–Cu(1)	92.5(3)
N(2)-Li(2)	2.048(8)	Li(2)–N(2)–Cu(1)	97.5(3)
N(3)-Li(2)	2.011(8)	Li(2)–N(3)–Cu(2)	97.3(2)
N(4)-Li(3)	1.995(8)	Li(3)–N(4)–Cu(2)	89.0(3)

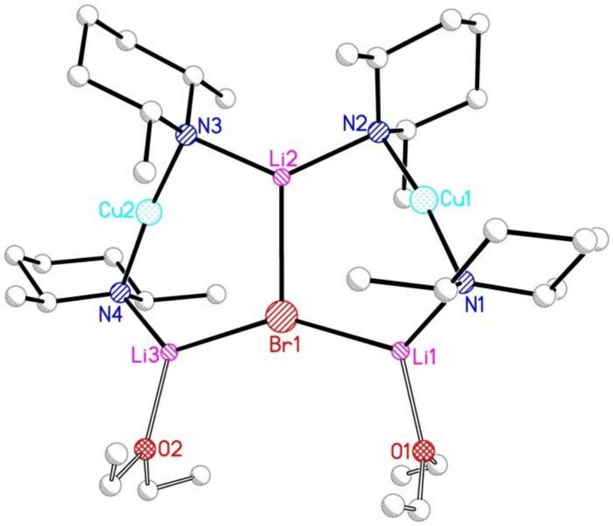

Figure S6. Adduct [{(DMP)$_2$CuLi(OEt$_2$)}$_2$LiBr] **6**. H atoms and minor ether disorder omitted for clarity.

1-9. Synthesis and characterization of 6´

*n*BuLi (2.5 mL, 1.6 M in hexanes, 4 mmol) was added dropwise to a solution of DMPH (0.54 ml, 4 mmol) in dry hexane (6 mL) and dry Et$_2$O (0.42 mL) under N$_2$ at –78 °C. The resulting solution of DMPLi was allowed to reach room temperature. It was then added to a suspension of copper(I) bromide (286 mg, 2 mmol) in dry hexane (3 mL) under N$_2$ at –78 °C. The mixture was allowed to reach room temperature whereupon it was filtered. Storage at –27 °C for 48 hours gave orange blocks of **6´**. Yield 171 mg (40 %), m. p. 93 ºC, dec. X-ray crystal data: $C_{37.65}H_{78.75}BrCu_2Li_3N_{4.55}O_{1.45}$, M = 846.34, triclinic, space group *P*–1, a = 12.6429(3), b = 12.9514(3), c = 16.6788(5) Å, α = 101.5700(10), β = 98.1560(10), γ = 114.4840(10) °, V = 2356.15(11) Å^3, Z = 2, ρ_{calcd} = 1.216 g cm^{-3}, Mo-K$_\alpha$ radiation, λ = 0.71070 Å, μ = 1.782 mm^{-1}, T = 180(2) K, 39087 data (10228 unique, R_{int} = 0.0637, θ < 27.04 °) were collected. $wR2 = \{\Sigma[w(F_o^2-F_c^2)^2]/\Sigma[w(F_o^2)^2]\}^{1/2}$ = 0.1586, conventional R = 0.0655 on F values of 5156 reflections with $F^2 > 2\sigma(F^2)$, S = 1.031, 530 parameters. Residual electron density extrema ±0.622 eÅ^{-3}.

^{1}H NMR (500 MHz, 298K, [D$_6$]benzene): δ 3.38 (q, 5.8H, ^{3}J(H,H) = 7 Hz; Et$_2$O), 3.26 – 2.65 (m, 8H; DMP-2,6), 2.52 (br, m, 1.1H; DMPH-2,6), 2.02-1.58 (m, 27.3H; DMP-3, 4, 5), 1.50 (br, m, 22.8H; DMP-Me), 1.18 (t, 8.4H, ^{3}J(H,H) = 7 Hz; Et$_2$O), 1.10 (d, 4.5H, DMP-Me), 0.87 (s, br, 1.6H; DMPH-NH)

^{13}C NMR (100 MHz, 298K, [D$_6$]benzene): δ 65.8 (Et$_2$O), 58.9, 57.7 (DMP-2,6), 52.7 (DMPH-2,6), 39.8, 39.5, 38.9 (DMP-3,5), 34.0 (DMPH-3,5), 28.9, 27.5, 27.0 (DMP-Me), 26.6 (DMP-4), 24.8 (DMPH-4), 23.0 (DMPH-Me), 15.2 (Et$_2$O)

^{7}Li NMR (194 MHz, 298K, [D$_6$]benzene): δ 2.16 (br, s, 0.4Li; TMPLi), 1.83 (br, s, 1Li; **6´**), 1.66 and 1.48 (s + sh, 2Li·L, L = 0.5DMPH + 1.5OEt$_2$; **6´**)

Elemental analysis calcd (%) for $C_{37.65}H_{78.75}BrCu_2Li_3N_{4.55}O_{1.45}$: C 53.43, H 9.38, N 7.53, Br 9.44; found: C 55.08, H 9.76, N 7.92, Br 9.94

Br(1)-Li(1)	2.499(9)	N(1)-Cu(1)-N(2)	174.17(18)
Br(1)-Li(2)	2.679(10)	N(3)-Cu(2)-N(4)	173.28(17)
Br(1)-Li(3)	2.496(9)	N(2)-Li(2)-N(3)	132.2(5)
Cu(1)-N(1)	1.895(4)	Li(1)–Br(1)–Li(2)	104.4(3)
Cu(1)-N(2)	1.894(4)	Li(3)–Br(1)–Li(2)	104.7(3)
Cu(2)-N(3)	1.896(4)	Br(1)–Li(1)–N(1)	122.1(4)
Cu(2)-N(4)	1.901(4)	Br(1)–Li(3)–N(4)	118.8(5)
N(1)-Li(1)	1.991(10)	Li(1)–N(1)–Cu(1)	90.6(3)
N(2)-Li(2)	2.032(9)	Li(2)–N(2)–Cu(1)	96.4(3)
N(3)-Li(2)	2.046(9)	Li(2)–N(3)–Cu(2)	95.5(3)
N(4)-Li(3)	2.004(10)	Li(3)–N(4)–Cu(2)	92.4(3)

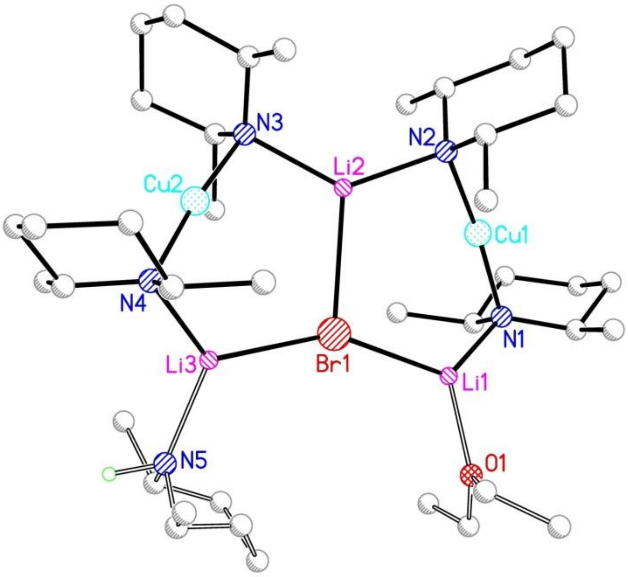

Figure S7. Adduct [{(DMP)$_2$CuLi(OEt$_2$)}$_{1.45}${(DMP)$_2$CuLi(DMPH)}$_{0.55}$LiBr] **6´**.
All H atoms (except NH) and minor Et$_2$O disorder omitted for clarity.

1-10. Synthesis and characterization of 7

*n*BuLi (1.25 mL, 1.6 M in hexanes, 2 mmol) was added dropwise to a solution of DMPH (0.27 mL, 2 mmol) in dry hexane (2 mL) and dry Et$_2$O (0.1 mL) under N$_2$ at –78 °C. The resulting solution of DMPLi was allowed to reach room temperature. It was then added to a suspension of copper(I) iodide (180 mg, 1 mmol) in dry hexane (2 mL) under N$_2$ at –78 °C. The mixture was allowed to reach room temperature whereupon it was filtered and the resulting pale yellow solution was stored at –27 °C yielding **7** as colourless blocks after 24 hours. Yield 56 mg (14 %) wrt CuI, m. p. 103 ºC dec. X-ray crystal data: $C_{36}H_{76}ICu_2Li_3N_4O_2$, M = 851.65, triclinic, space group P–1, a = 12.733(3), b = 12.961(3), c = 15.777(3) Å, α = 78.34(2), β = 88.76(3), γ = 63.77(3) °, V = 2280.5(8) Å^3, Z = 2, ρ_{calcd} = 1.240 g cm^{-3}, Mo-K$_\alpha$ radiation, λ = 0.71073 Å, μ = 1.639 mm^{-1}, T = 173(2) K. 22384 data (8031 unique, R_{int} = 0.0570, θ < 25.38 °) were collected. $wR2 = \{\Sigma[w(F_o^2-F_c^2)^2]/\Sigma[w(F_o^2)^2]\}^{1/2}$ = 0.2205, conventional

R = 0.0789 on *F* values of 5723 reflections with $F^2 > 2\sigma(F^2)$, *S* = 1.057, 455 parameters. Residual electron density extrema ±1.572 eÅ^{-3}.

^{1}H NMR (500 MHz, 298K, [D$_6$]benzene): δ 3.40 (q, 8H, ^{3}J(H,H) = 7 Hz; Et$_2$O), 3.05 – 2.70 (br, m, 8H; DMP-2,6), 2.01-1.60 (br, m, 24H; DMP-3,4,5), 1.53 – 1.26 (br, m, 18H; DMP-Me), 1.12 (t, 12H, ^{3}J(H,H) = 7 Hz; Et$_2$O), 1.10 (d, 6H; DMP-Me), 0.90 (s, br, 1.4H; DMPH-NH)

^{13}C NMR (100 MHz, 298K, [D$_6$]benzene): δ 65.9 (Et$_2$O), 58.8 (DMP-2,6), 52.8 (DMPH-2,6), 39.6 (DMP-3,5), 33.9 (DMPH-3,5), 27.4 (DMP-Me), 26.4 (DMPH-4), 24.7 (DMP-4), 23.1 (DMPH-Me), 15.1 (Et$_2$O)

^{7}Li NMR (194 MHz, 298K, [D$_6$]benzene): δ 2.17 (br, s, 0.2Li; TMPLi), 1.84 (br, s, 1Li; **7**), 1.41 (br, s, 2Li·OEt$_2$;**7**)

Elemental analysis calcd (%) for $C_{36}H_{76}ICu_2Li_3N_4O_2$: C 49.60, H 8.62, N 6.17, I 14.56; found: C 48.59, H 8.62, N 6.43, I 14.83

I(1A)-Li(1)	2.720(13)	N(1)-Cu(1)-N(2)	177.0(2)
I(1A)-Li(2)	2.971(16)	N(3)-Cu(2)-N(4)	176.0(3)
I(1A)-Li(3)	2.667(14)	N(2)-Li(2)-N(3)	133.5(8)
Cu(1)-N(1)	1.922(6)	Li(1)–I(1A)–Li(2)	102.0(4)
Cu(1)-N(2)	1.910(6)	Li(3)–I(1A)–Li(2)	102.1(4)
Cu(2)-N(3)	1.908(6)	I(1A)–Li(1)–N(1)	124.6(6)
Cu(2)-N(4)	1.918(6)	I(1A)–Li(3)–N(4)	120.3(6)
N(1)-Li(1)	1.916(13)	Li(1)–N(1)–Cu(1)	96.1(5)
N(2)-Li(2)	2.022(13)	Li(2)–N(2)–Cu(1)	101.2(5)
N(3)-Li(2)	2.000(14)	Li(2)–N(3)–Cu(2)	100.1(5)
N(4)-Li(3)	1.934(14)	Li(3)–N(4)–Cu(2)	96.8(5)

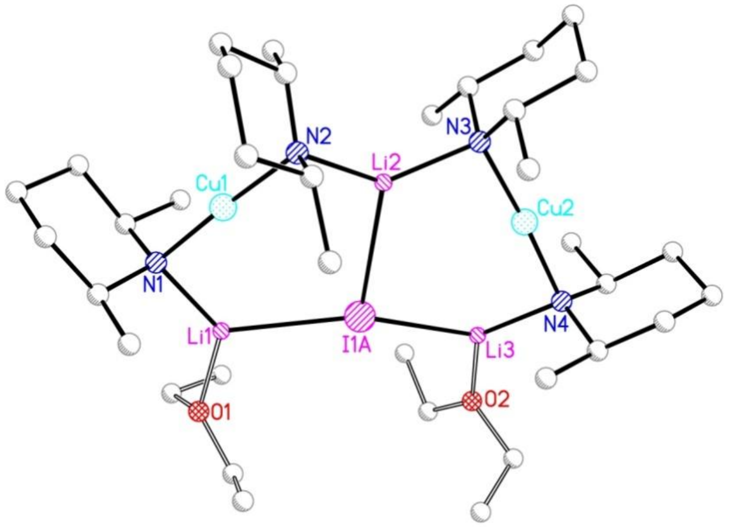

Figure S8. Adduct [{(DMP)$_2$CuLi(OEt$_2$)}$_2$LiI]**7**. H atoms and minor iodide disorder omitted for clarity.

Synthesis and characterization of 8

a) *n*BuLi (1.25 mL, 1.6 M in hexanes, 4 mmol) was added dropwise to a solution of DMPH (0.54 mL, 4 mmol) in dry toluene (5 mL) under N_2 at –78 °C. The resulting solution of DMPLi was allowed to reach room temperature. It was then added to a suspension of copper(I) bromide (286 mg, 2 mmol) in dry toluene (2 mL) under N_2 at –78 °C. The solvent was removed and dry THF (8 mL) was added, whereupon the mixture was filtered. The resulting solution was reduced to *ca*. 3 mL volume and 12 mL hexane was added. The orange-red solution was stored at –27 °C yielding **8** as fine, colourless needles after 24 hours. Yield 215 mg (22 %) wrt CuBr, m. p. 81 ºC dec. X-ray crystal data: $C_{14.67}H_{29.33}Br_{0.33}Cu_{0.67}LiN_{1.33}O_{1.33}$, M = 321.66, triclinic, space group P–1, a = 12.234(2), b = 14.798(3), c = 17.745(4) Å, α = 112.84(3), β = 91.90(3), γ = 113.92(3) °, V = 2636.4(9) Å^3, Z = 6, ρ_{calcd} = 1.216 g cm^{-3}, Mo-K$_\alpha$ radiation, λ = 0.71073 Å, μ = 1.603 mm^{-1}, T = 180(2) K. 38698 data (9608 unique, R_{int} = 0.1630, θ < 25.44 °) were collected. $wR2 = \{\Sigma[w(F_o^2-F_c^2)^2]/\Sigma[w(F_o^2)^2]\}^{1/2}$ = 0.1162, conventional R = 0.0734 on F values of 4462 reflections with $F^2 > 2\sigma(F^2)$, S = 1.016, 531 parameters. Residual electron density extrema ±0.397 eÅ^{-3}.

^{1}H NMR (500 MHz, 298K, [D$_6$]benzene): *δ* 3.67 (m, 16H; THF), 3.19 – 2.74 (br, m, 8H; DMP-2,6), 2.25 (br, m, 4H; DMP-3), 2.07 – 1.76 (br, m, 16H; DMP-3,4,5), 1.63 (br, m, 23H; DMP-Me), 1.50 (m, 4H; DMP-4,5), 1.42 (m, 16H; THF), 1.09 (d, 1H; DMP-Me), 0.88 (br, s, 0.2H; DMPH-NH)

^{13}C NMR (125 MHz, 298K, [D$_6$]benzene): *δ* 68.2 (THF), 59.0 (DMP-2,6), 52.4 (DMPH-2,6), 39.7 (DMP-3,5), 34.2 (DMPH-3,5), 27.5 (DMP-Me), 26.8 (DMP-4), 25.3 (THF), 23.1 (DMPH-Me)

^{7}Li NMR (194 MHz, 298K, [D$_6$]benzene): *δ* 1.52 (br, s, 3Li; **8**)

Elemental analysis calcd (%) for $C_{14.67}H_{29.33}Br_{0.33}Cu_{0.67}LiN_{1.33}O_{1.33}$: Br 8.28; found: Br 8.13. It was not possible to collect satisfactory C, H and N data for this compound despite repeated attempts.

b) *n*BuLi (1.25 mL, 1.6 M in hexanes, 4 mmol) was added dropwise to a solution of DMPH (0.54 mL, 4 mmol) in dry toluene (5 mL) under N_2 at –78 °C. The resulting solution of DMPLi was allowed to reach room temperature. It was then added to a suspension of copper(I) bromide (286 mg, 2 mmol) in dry toluene (2 mL) under N_2 at –78 °C. The solvent was removed and dry THF (8 mL) was added, whereupon the mixture was filtered. The resulting solution was reduced to *ca*. 2 mL volume. The orange-brown solution was stored at –27 °C yielding **8** as colourless blocks after 48 hours. Yield 246 mg (26 %) wrt CuBr, m.p. 80 °C. A crystallographic cell check verified the crystal data obtained by route a.

^{1}H NMR (500 MHz, 298K, [D$_6$]benzene): *δ* 3.68 (m, 16H; THF), 3.27 – 2.65 (br, m, 8H; DMP-2,6), 2.25 (br, m, 4H; DMP-3), 2.07 – 1.76 (br, m, 16H; DMP-3,4,5), 1.66 (br, m, 23H; DMP-Me), 1.50 (m, 4H; DMP-4,5), 1.42 (m, 16H; THF), 1.09 (d, 1H; DMP-Me), 0.88 (br, s, 0.2H; DMPH-NH)

^{13}C NMR (125 MHz, 298K, [D$_6$]benzene): *δ* 68.2 (THF), 59.1 (DMP-2,6), 52.5 (DMPH-2,6), 39.7 (DMP-3,5), 34.2 (DMPH-3,5), 27.5 (DMP-Me), 27.3 (DMP-4), 25.3 (THF), 25.1 (DMPH-4), 23.1 (DMPH-Me)

^{7}Li NMR (194 MHz, 298K, [D$_6$]benzene): *δ* 1.55 (br, s, 3Li; **8**)

Elemental analysis calcd (%) for $C_{14.67}H_{29.33}Br_{0.33}Cu_{0.67}LiN_{1.33}O_{1.33}$: C 54.76, H 9.19, N 5.81, Br 8.28; found: C 53.93, H 8.97, N 5.85, Br 7.94

Br(1)-Li(1)	2.609(11)	N(1)-Cu(1)-N(2)	177.2(2)
Br(1)-Li(2)	2.677(11)	N(3)-Cu(2)-N(4)	176.1(2)
Br(1)-Li(3)	2.602(12)	N(2)-Li(2)-N(3)	132.2(6)
Cu(1)-N(1)	1.903(5)	Li(1)–Br(1)–Li(2)	107.5(3)
Cu(1)-N(2)	1.904(5)	Li(3)–Br(1)–Li(2)	105.2(4)
Cu(2)-N(3)	1.882(5)	Br(1)–Li(1)–N(1)	115.4(5)
Cu(2)-N(4)	1.904(6)	Br(1)–Li(3)–N(4)	116.6(6)
N(1)-Li(1)	2.067(13)	Li(1)–N(1)–Cu(1)	94.3(4)
N(2)-Li(2)	2.045(11)	Li(2)–N(2)–Cu(1)	93.9(4)
N(3)-Li(2)	2.029(11)	Li(2)–N(3)–Cu(2)	94.2(4)
N(4)-Li(3)	2.094(15)	Li(3)–N(4)–Cu(2)	92.0(4)

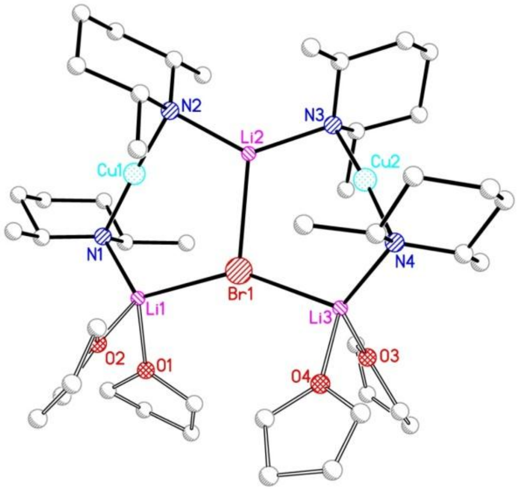

Figure S9. Adduct [{(DMP)$_2$CuLi(THF)$_2$}$_2$LiBr] **8**. H atoms omitted for clarity.

2. Directed *ortho* Cupration reactions

2-1. Preparation of 2-iodo-*N*,*N*-diisopropylbenzamide using a Lipshutz formulation base

*n*BuLi (10.00 mL, 1.6 M in hexanes, 16.0 mmol) was added to a solution of 2,6-*cis*-dimethylpiperidine (2.16 mL, 16.0 mmol) in dry THF (6.0 mL) under N_2 at –78 °C. The resulting solution of DMPLi was then added to a suspension of copper(I) bromide (1.14 g, 8.0 mmol) in dry THF (4 mL) at –78 °C. The resulting black slurry was allowed to reach room temperature whereupon it was filtered. This resulting dark orange solution was treated with a solution of *N*,*N*-diisopropylbenzamide (0.82 g, 4 mmol) in THF (5.0 mL) at –78 ºC. The reaction pot was returned to 0 °C and stirred for 3 hours. whereupon it was cooled once again to –78 °C and I_2 (2.02 g, 8 mmol) was introduced as a THF solution (10 mL). The mixture was allowed to reach room temperature at which point it was stirred for 16 hours. The reaction mixture was poured into sat. $NaHCO_3$, sat. NH_4Cl (50 mL each) and sat. $Na_2S_2O_3$ (200 mL) and extracted with AcOEt. The AcOEt layer was washed with brine, dried ($MgSO_4$) and the solvent removed *in vacuo*. Purification by silica gel flash chromatography gave 2-iodo-*N*,*N*-diisopropylbenzamide as a yellow-white solid (1.06 g, 3.20 mmol, 80%), m.p. 188-189 ºC.

^{1}H NMR (400 MHz, [D]chloroform): δ 7.81 (dd, 1H; Ar), 7.35 (dt., 1H; Ar), 7.13 (dd, 1H; Ar), 7.02 (dt, 1H; Ar), 3.58 (sept, 1H; NCH), 3.51 (sept, 1H; NCH), 1.60 (d, 3H; Me), 1.56 (d, 3H; Me), 1.27 (d, 3H; Me), 1.06 (d, 3H; Me)

^{13}C NMR (100 MHz, [D]chloroform): δ 169.8 (CO), 144.3 (1-Ar), 139.3, 129.4, 128.2, 125.8 (Ar), 92.3 (2-Ar), 51.2, 46.0 (NC), 20.8, 20.7, 20.1 (Me)

2-2. Preparation of 2-iodo-*N*,*N*-diisopropylbenzamide using pre-isolated 6

Crystals of **6** were prepared via route b, and subsequently isolated after storage at –30 °C for at least 24 hours. 1.15g (1.4 mmol) of **6** were placed in a Schlenk tube and dissolved in THF (7 mL). This was treated with a solution of *N*,*N*-diisopropylbenzamide (0.29 g, 1.4 mmol) in THF (5.0 mL) at –78 ºC. The reaction pot was returned to 0 °C and stirred for 3 hours. whereupon it was cooled once again to –78 °C and I_2 (0.72 g, 2.8 mmol) was introduced as a THF solution (10 mL). The mixture was allowed to reach room temperature at which point it was stirred for 16 hours. Work-up was as above (Section 2-1.) and yielded 2-iodo-*N*,*N*-diisopropylbenzamide as a yellow-white solid (0.38 g, 1.15 mmol, 82%), m.p. 185-186 ºC.

^{1}H NMR (400 MHz, [D]chloroform): δ 7.81 (dd, 1H; Ar), 7.35 (dt., 1H; Ar), 7.14 (dd, 1H; Ar), 7.03 (dt, 1H; Ar), 3.58 (sept, 1H; NCH), 3.52 (sept, 1H; NCH), 1.60 (d, 3H; Me), 1.56 (d, 3H; Me), 1.27 (d, 3H; Me), 1.06 (d, 3H; Me)

^{13}C NMR (100 MHz, [D]chloroform): δ 169.9 (CO), 144.3 (1-Ar), 139.3, 129.6, 128.3, 126.0 (Ar), 92.3 (2-Ar), 51.4, 46.1 (NC), 20.9, 20.8, 20.2 (Me)

3. Computational Section

3-1. Details of computational methods

All calculations were carried with the Gaussian 09 program package.[5] The molecular structures and harmonic vibrational frequencies were obtained using the hybrid density functional method based on Becke's three-parameter exchange function and the Lee-Yang-Parr nonlocal correlation functional (B3LYP).[6] Ahlrichs' SVP[7] all-electron basis set was used for Cu atoms and 6-31+G* for all other atoms (denoted as 631SVP in the text). Geometry optimization and vibrational analysis were performed at the same level. All stationary points were optimized without any symmetry assumptions, and characterized by normal coordinate analysis at the same level of theory (number of imaginary frequencies, NIMAG, 0 for minima and 1 for TSs). The intrinsic reaction coordinate (IRC) method was used to track minimum energy paths from transition structures to the corresponding local minima.[8]

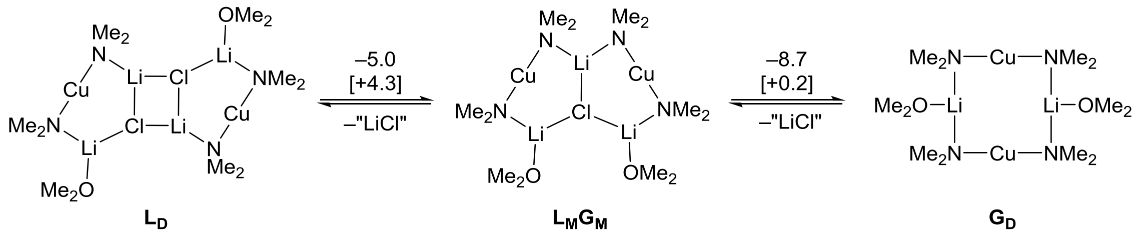

Scheme S1 (Scheme 3 in the paper). The interconversion of Lipshutz- and Gilman-type dimers at B3LYP/631SVP level (ΔE [ΔG] are in kcal mol^{-1}). "LiCl" is 1/4[LiCl(OMe_2)]$_4$ – OMe_2.

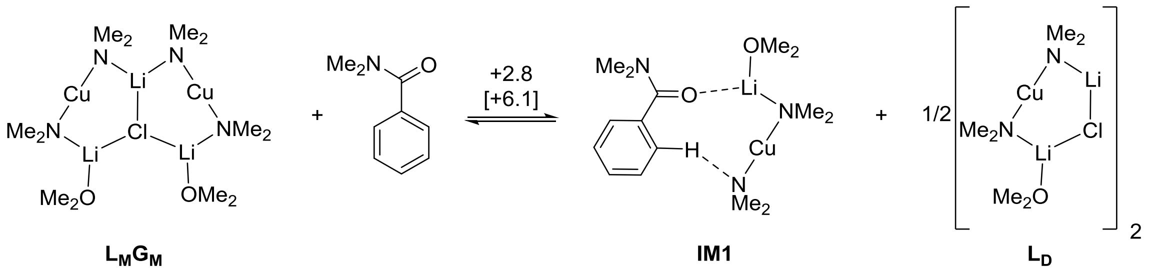

Scheme S2. The interconversion of Lipshutz-Gilman adduct $\mathbf{L_M G_M}$ and the monomeric Gilman-(*N*,*N*-dimethylbenzamide) complex (**IM1**) for DoC at B3LYP/631SVP level (ΔE [ΔG] are in kcal mol^{-1}).

3-2. Cartesian coordinates and energies

[LiCl(Me$_2$O)]$_4$

Energy (B3LYP) = –2491.63284631 A.U.

Gibbs Free Energy = –2491.374117 A.U.

Li	0.144108	1.632149	-0.758491
Cl	-1.365014	1.502332	1.071193
Li	0.452706	0.139974	1.799389
Cl	2.128108	0.709258	0.153185
Li	1.091855	-1.209768	-0.777132
Cl	-0.628334	-0.195873	-2.088599
Li	-1.750886	-0.584194	-0.017922
Cl	-0.185343	-2.032809	1.048618
O	2.268391	-2.476475	-1.640981
C	3.355612	-2.033696	-2.448964
C	1.821296	-3.792996	-1.953300
H	4.216061	-2.706697	-2.329971
H	3.621517	-1.033147	-2.102221
H	3.061467	-1.996762	-3.507196
H	1.012935	-4.027389	-1.257805
H	2.640549	-4.513524	-1.822460
H	1.450467	-3.841352	-2.986603
O	0.990925	0.308891	3.640237
C	1.837503	-0.668367	4.240768
C	1.062685	1.589816	4.261955
H	2.890929	-0.364685	4.167581
H	1.682536	-1.601622	3.695659
H	1.566479	-0.807629	5.296419
H	0.365766	2.240839	3.730395
H	2.080713	1.997225	4.191475
H	0.767642	1.515865	5.317764
O	-3.580878	-1.194104	-0.015894
C	-3.917884	-2.564587	-0.211428
C	-4.671631	-0.299664	-0.216194
H	-4.720673	-2.860875	0.477854
H	-3.019204	-3.146477	0.002430
H	-4.239905	-2.739634	-1.247518
H	-4.301110	0.705472	-0.005453
H	-5.492856	-0.540971	0.472795
H	-5.032761	-0.356097	-1.252642
O	0.319628	3.359657	-1.607081
C	-0.831884	4.140230	-1.915851
C	1.453608	3.667418	-2.413524
H	-0.614183	5.208738	-1.780372
H	-1.617640	3.835758	-1.221604
H	-1.156732	3.959070	-2.949872
H	2.269667	3.028509	-2.070178
H	1.733151	4.722787	-2.288937
H	1.241397	3.466015	-3.472836

Dimethyl ether (Me$_2$O, solvent model = *S*)

Energy (B3LYP) = –155.0314738 A.U.

Gibbs Free Energy = –154.9768328 A.U.

O	0.000001	-0.586396	0.000004
C	-1.177266	0.195215	0.000000
C	1.177265	0.195214	0.000000
H	-1.234746	0.835574	0.895679
H	-2.023006	-0.497181	-0.000239
H	-1.234507	0.835902	-0.895458
H	2.023008	-0.497177	-0.000234
H	1.234743	0.835577	0.895677
H	1.234508	0.835901	-0.895459

***N,N*-Dimethylbenzamide**

Energy (B3LYP) = –479.5750019 A.U.

Gibbs Free Energy = –479.4276779 A.U.

C	0.446799	0.237644	-0.084615
C	1.327793	1.139727	0.529713
C	2.693613	0.859025	0.593862
C	3.197581	-0.312370	0.018377
C	2.328718	-1.202608	-0.619873
C	0.958326	-0.932682	-0.665157
H	0.932134	2.062702	0.943204
H	3.366463	1.559069	1.082511
H	4.262781	-0.525450	0.058783
H	2.717138	-2.104592	-1.086043
H	0.288357	-1.622538	-1.172000
C	-1.003649	0.628851	-0.203262
N	-1.965660	-0.320593	0.057365

C	-1.769390	-1.493899	0.901959
H	-2.379667	-1.404675	1.812328
H	-2.073844	-2.408008	0.375227
H	-0.726074	-1.589159	1.199024
C	-3.359528	0.036807	-0.184614
H	-3.911069	-0.861451	-0.484380
H	-3.829046	0.453029	0.718798
H	-3.409961	0.784785	-0.975708
O	-1.299896	1.767183	-0.573955

Lipshutz dimer (L_D)

Energy (B3LYP) = –5080.03598750 A.U.

Gibbs Free Energy = –5079.623995 A.U.

Cu	3.410659	-1.787810	0.004344
Li	3.596013	0.958833	0.165558
Li	0.744881	-1.311840	0.261467
Cl	1.331117	0.944106	0.743411
N	4.756090	-0.493801	-0.485466
N	1.903071	-2.855380	0.539663
C	5.012588	-0.441004	-1.926791
C	6.024306	-0.699474	0.216667
C	1.952955	-3.285504	1.939239
C	1.634071	-4.015908	-0.310388
H	5.707172	0.383614	-2.186636
H	4.080435	-0.274877	-2.482140
H	5.471252	-1.366285	-2.323950
H	6.747871	0.112613	0.000429
H	6.530263	-1.642791	-0.063080
H	5.868125	-0.721528	1.302724
H	1.000634	-3.759973	2.247939
H	2.750056	-4.026689	2.140721
H	2.126794	-2.428396	2.602634
H	0.675995	-4.500189	-0.037348
H	1.565070	-3.717034	-1.363744
H	2.413308	-4.798661	-0.237845
O	4.248043	2.778794	0.364397
C	5.538220	3.173682	-0.099404
C	3.511989	3.856888	0.948410
H	6.012518	2.289891	-0.528308
H	6.146661	3.551333	0.733766
H	5.446611	3.954313	-0.867005
H	2.546747	3.454887	1.257404
H	3.363056	4.657726	0.211673
H	4.050787	4.255333	1.818832
Cu	-3.410727	1.787643	-0.003282
Li	-3.595792	-0.958788	-0.166116
Li	-0.744944	1.312425	-0.261926
Cl	-1.331087	-0.943551	-0.744380
N	-4.755471	0.493193	0.487212
N	-1.903746	2.855659	-0.539455
C	-6.024521	0.699349	-0.213276
C	-5.010282	0.439256	1.928791
C	-1.634823	4.016523	0.310148
C	-1.954180	3.285322	-1.939155
H	-6.747796	-0.112941	0.003174
H	-5.869612	0.722261	-1.299495
H	-6.530193	1.642419	0.067811
H	-5.704478	-0.385635	2.188799
H	-5.468569	1.364186	2.327203
H	-4.077463	0.272791	2.482921
H	-0.676953	4.500982	0.036698
H	-2.414292	4.799040	0.237560
H	-1.565457	3.717998	1.363578
H	-1.002066	3.759901	-2.248332
H	-2.128068	2.427965	-2.602216
H	-2.751508	4.026273	-2.140609
O	-4.248104	-2.778431	-0.366984
C	-5.537790	-3.173910	0.097679
C	-3.512364	-3.855933	-0.952469
H	-6.011832	-2.290548	0.527748
H	-5.445353	-3.955162	0.864550
H	-6.146935	-3.550969	-0.735244
H	-2.547477	-3.453505	-1.262023
H	-4.051870	-4.253835	-1.822700
H	-3.362589	-4.657297	-0.216473

Lipshutz-Gilman adduct (L_MG_M)

Energy (B3LYP) = –4612.16718224 A.U.

Gibbs Free Energy = –4611.750396 A.U.

Cu	2.778772	-1.134540	0.200393
Li	2.271570	1.430838	0.009272
Li	0.000228	-1.667337	-0.000573
Cl	-0.000337	0.932023	-0.001037
N	3.610399	0.412631	1.007545
N	1.705205	-2.469429	-0.653441
C	5.035569	0.546017	0.713193

C	3.414557	0.487388	2.457805
C	1.930458	-3.836356	-0.180701
C	1.775381	-2.452219	-2.115562
H	5.442114	1.505920	1.093542
H	5.213873	0.511978	-0.369224
H	5.652555	-0.250070	1.171718
H	3.779863	1.451470	2.865029
H	3.949083	-0.307009	3.012872
H	2.349579	0.401596	2.708563
H	1.181280	-4.535280	-0.598497
H	2.924690	-4.235813	-0.460524
H	1.852830	-3.885180	0.912648
H	1.021393	-3.129935	-2.562562
H	1.582639	-1.442296	-2.501209
H	2.757374	-2.776040	-2.513186
O	2.666676	2.888565	-1.209928
C	1.780243	3.305638	-2.249943
C	3.918736	3.572538	-1.235157
H	0.860840	2.730258	-2.133831
H	2.226443	3.105991	-3.233630
H	1.563855	4.378850	-2.157737
H	4.519980	3.178351	-0.414675
H	3.766689	4.652245	-1.100183
H	4.435242	3.393439	-2.187967
Cu	-2.778752	-1.134983	-0.199634
Li	-2.272142	1.431001	-0.009738
N	-3.611622	0.411888	-1.006209
N	-1.704200	-2.469782	0.653111
C	-5.036515	0.544872	-0.710250
C	-3.417568	0.486044	-2.456750
C	-1.929076	-3.836614	0.179915
C	-1.773638	-2.453197	2.115269
H	-5.443826	1.504556	-1.090337
H	-5.213568	0.511015	0.372377
H	-5.653733	-0.251526	-1.167911
H	-3.783553	1.449875	-2.863963
H	-3.952637	-0.308704	-3.010786
H	-2.352884	0.400351	-2.708775
H	-1.179419	-4.535390	0.597101
H	-2.923016	-4.236621	0.460001
H	-1.851913	-3.884967	-0.913491
H	-1.019076	-3.130699	2.561625
H	-1.581230	-1.443325	2.501213
H	-2.755266	-2.777681	2.513253
O	-2.666364	2.889728	1.208705
C	-1.779007	3.307716	2.247576
C	-3.918578	3.573371	1.234722
H	-0.859452	2.732739	2.130691
H	-2.224023	3.108258	3.231837
H	-1.563282	4.380998	2.154662
H	-4.520505	3.178490	0.415073
H	-3.766965	4.653033	1.098906
H	-4.434126	3.394736	2.188141

Gilman dimer (G_D)

Energy (B3LYP) = –4144.30428195 A.U.

Gibbs Free Energy = –4143.883362 A.U.

O	-3.331807	0.104064	0.978975
C	-4.722148	-0.118770	0.773729
H	-5.118242	-0.813120	1.527985
H	-5.278260	0.828191	0.821588
H	-4.838046	-0.561710	-0.217698
C	-3.037616	0.652934	2.259558
H	-3.539291	1.621853	2.392411
H	-3.355708	-0.035513	3.054989
H	-1.956183	0.795127	2.309777
Li	-2.018823	-0.008533	-0.541215
N	-1.602766	-1.887693	-1.027773
Cu	0.046127	1.873817	-0.107611
Cu	-0.046354	-1.873408	0.107380
C	-1.312229	-2.110015	-2.444518
H	-0.907454	-3.119065	-2.657278
H	-2.224050	-2.003508	-3.064439
H	-0.582576	-1.377959	-2.811276
C	-2.599418	-2.868652	-0.595242
H	-2.853586	-2.721619	0.461375
H	-3.535801	-2.777721	-1.182699
H	-2.264190	-3.917465	-0.713006
N	1.603221	1.888087	1.026633
C	1.313504	2.111448	2.443389
H	0.583772	1.379911	2.811026
H	2.225615	2.005008	3.062897
H	0.909223	3.120789	2.655691
C	2.599993	2.868421	0.592935
H	2.853607	2.720587	-0.463702
H	2.265179	3.917427	0.710160
H	3.536628	2.777576	1.180003
Li	2.018581	0.008587	0.540843

O	3.332195	-0.105242	-0.978592
C	4.722557	0.116335	-0.772100
H	4.837876	0.559902	0.219112
H	5.277762	-0.831220	-0.818684
H	5.120113	0.809727	-1.526467
C	3.038746	-0.654601	-2.259134
H	3.539473	-1.624165	-2.390852
H	1.957209	-0.795608	-2.310398
H	3.358448	0.032991	-3.054660
N	1.495147	-1.768343	1.254671
C	2.487425	-2.814768	1.005795
H	3.408984	-2.645818	1.598454
H	2.131485	-3.829667	1.269868
H	2.774315	-2.835189	-0.052695
C	1.157561	-1.773523	2.679103
H	2.049433	-1.577091	3.306142
H	0.419755	-0.994603	2.905745
H	0.738323	-2.738047	3.026808
N	-1.496046	1.768971	-1.254055
C	-1.159171	1.775162	-2.678658
H	-0.421359	0.996511	-2.906189
H	-2.051328	1.579012	-3.305380
H	-0.740263	2.739984	-3.025929
C	-2.488461	2.814988	-1.004018
H	-3.410302	2.646148	-1.596270
H	-2.774774	2.834702	0.054641
H	-2.132929	3.830135	-1.267684

IM1

Energy (B3LYP) = –2551.71974767 A.U.

Gibbs Free Energy = –2551.356388 A.U.

Li	-1.335710	1.292483	0.456942
Cu	-1.730929	-1.184379	-0.518313
C	-0.071374	-3.551422	-0.600821
N	-0.401800	-2.303279	-1.254115
C	-0.335516	-2.470543	-2.691937
O	-1.261419	3.149103	-0.145019
C	-2.389627	3.901134	-0.585479
C	-0.039732	3.884018	-0.210494
C	3.358928	1.294461	0.520774
C	4.300028	1.576190	-0.473612
C	4.196672	0.963675	-1.726992
C	3.151577	0.069739	-1.982644
C	2.204036	-0.221840	-0.995792
C	2.316668	0.393827	0.259359
C	1.263258	0.146414	1.306649
O	0.269970	0.906652	1.366057
N	1.431803	-0.867723	2.180020
C	0.406866	-1.142097	3.186221
C	2.512004	-1.849726	2.101910
H	-0.104562	-3.443295	0.492647
H	0.952464	-3.897364	-0.868058
H	-0.751072	-4.393605	-0.860436
H	-0.546800	-1.523632	-3.206632
H	-1.047693	-3.225591	-3.092150
H	0.674052	-2.805876	-3.019308
H	-2.262565	4.207267	-1.633165
H	-2.522498	4.794511	0.040686
H	-3.263878	3.255215	-0.496409
H	0.170775	4.184670	-1.246079
H	0.748791	3.223857	0.151634
H	-0.098639	4.779164	0.424466
H	3.437021	1.770109	1.496228
H	5.110470	2.271373	-0.269069
H	4.929431	1.181871	-2.500014
H	3.069960	-0.407130	-2.956088
H	-0.221196	-1.984695	2.871486
H	-0.218367	-0.260355	3.318288
H	0.894605	-1.395732	4.133446
H	3.011488	-1.921952	3.075318
H	2.103908	-2.832584	1.837758
H	3.240634	-1.558469	1.347765
H	1.378305	-0.920126	-1.177511
N	-2.907542	0.140445	0.275344
C	-3.461450	-0.266648	1.567049
H	-4.055042	0.548738	2.028402
H	-4.135636	-1.142708	1.499679
H	-2.658857	-0.528034	2.268891
C	-4.010563	0.508161	-0.609506
H	-4.715523	-0.324011	-0.799928
H	-4.619158	1.332676	-0.181932
H	-3.632628	0.841036	-1.584829

----

4. References

1. T. Kottke, D. Stalke, *J. Appl. Cryst*. **1993**, *26*, 615-619
2. G. M. Sheldrick, *Acta Crystallogr., Sect. A*, **1990**, *46*, 467-473.
3. G. M. Sheldrick, *SHELXL-97 Program for Crystal Structure Refinement*, University of Göttingen, Germany, **1997**.
4. R. H. Blessing, *Acta Crystallogr., Sect. A*, **1995**, *51*, 33-38.
5. Gaussian 09, Revision C.01, M. J. Frisch, G. W. Trucks, H. B. Schlegel, G. E. Scuseria, M. A. Robb, J. R. Cheeseman, G. Scalmani, V. Barone, B. Mennucci, G. A. Petersson, H. Nakatsuji, M. Caricato, X. Li, H. P. Hratchian, A. F. Izmaylov, J. Bloino, G. Zheng, J. L. Sonnenberg, M. Hada, M. Ehara, K. Toyota, R. Fukuda, J. Hasegawa, M. Ishida, T. Nakajima, Y. Honda, O. Kitao, H. Nakai, T. Vreven, J. A. Montgomery, Jr., J. E. Peralta, F. Ogliaro, M. Bearpark, J. J. Heyd, E. Brothers, K. N. Kudin, V. N. Staroverov, R. Kobayashi, J. Normand, K. Raghavachari, A. Rendell, J. C. Burant, S. S. Iyengar, J. Tomasi, M. Cossi, N. Rega, J. M. Millam, M. Klene, J. E. Knox, J. B. Cross, V. Bakken, C. Adamo, J. Jaramillo, R. Gomperts, R. E. Stratmann, O. Yazyev, A. J. Austin, R. Cammi, C. Pomelli, J. W. Ochterski, R. L. Martin, K. Morokuma, V. G. Zakrzewski, G. A. Voth, P. Salvador, J. J. Dannenberg, S. Dapprich, A. D. Daniels, Ö. Farkas, J. B. Foresman, J. V. Ortiz, J. Cioslowski, D. J. Fox, Gaussian, Inc., Wallingford CT, **2010**.
6. (a) A. D. Becke, *Phys. Rev.* **1988**, *A38*, 3098-3100; (b) A. D. Becke, *J. Chem. Phys.* **1993**, *98*, 1372-1377; (c) A. D. Becke, *J. Chem. Phys.* **1993**, *98*, 5648-5652; (d) C. Lee, W. Yang, R. G. Parr, *Phys. Rev.* **1988,** *B37*, 785-788.
7. A. Schäfer, H. Horn, R. Ahlrichs, *J. Chem. Phys*. **1992**, *97*, 2571-2577.
8. (a) K. Fukui, *Acc. Chem. Res.* **1981**, *14*, 363-368; (b) K. Ishida, K. Morokuma, A. Komornicki, *J. Chem. Phys.* **1977**, *66*, 2153-2156; (c) C. Gonzalez, H. B. Schlegel, *J. Chem. Phys.* **1989**, *90*, 2154-2161. (d) H. B. Schlegel, C. Gonzalez, *J. Phys. Chem.* **1990**, *94*, 5523-5527.
